# Structure Characterization of a Disordered Peptide Using In-Droplet Hydrogen/Deuterium Exchange Mass Spectrometry and Molecular Dynamics

**DOI:** 10.1021/acsphyschemau.4c00048

**Published:** 2024-11-13

**Authors:** Mohammad A. Rahman, Mst Nigar Sultana, Daud Sharif, Sultan Mahmud, Justin Legleiter, Peng Li, Blake Mertz, Stephen J. Valentine

**Affiliations:** † Department of Chemistry, West Virginia University, Morgantown, West Virginia 26506, United States; ‡ Department of Biochemistry & Molecular Biology, University of Nevada, Reno, Reno, Nevada 89557, United States; § Alivexis, Cambridge, Massachusetts 02142, United States

**Keywords:** reactivity prediction, high-throughput HDX, intrinsically disordered protein, in-droplet reactions, mass spectrometry

## Abstract

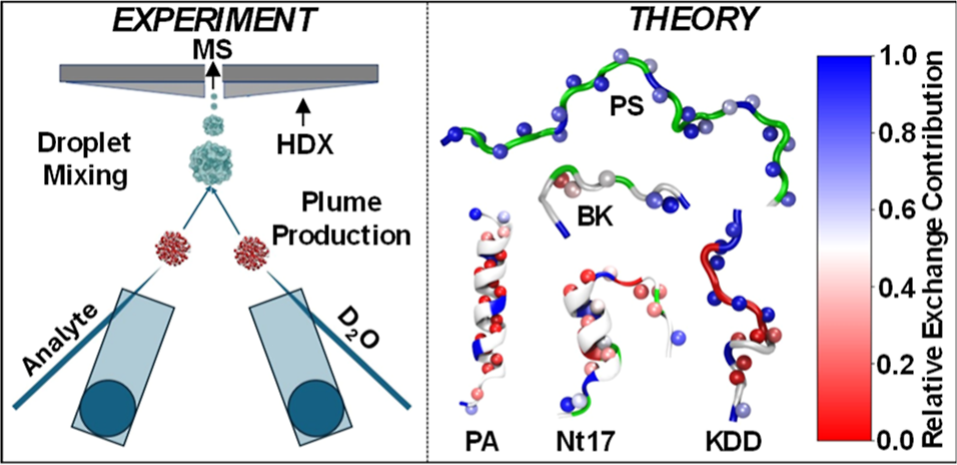

In-droplet hydrogen/deuterium exchange (HDX)-mass spectrometry (MS) experiments have been conducted for peptides of highly varied conformational type. A new model is presented that combines the use of protection factors (PF) from molecular dynamics (MD) simulations with intrinsic HDX rates (*k*_int_) to obtain a structure-to-reactivity calibration curve. Using the model, the relationship of peptide structural flexibility and HDX reactivity for different peptides is elucidated. Additionally, the model is used to describe the degree of conformational flexibility and structural bias for the disease-relevant Nt17 peptide; although highly flexible, intrinsically primed for facile conversion to α-helical conformation upon binding with molecular partners imparts significant in-droplet HDX protection for this peptide. In the future, a scale may be developed whereby HDX reactivity is predictive of the degree of structural flexibility and bias (propensity to form 2° structural elements such as α-helix, β-sheet, and β-turn) for intrinsically disordered regions (IDRs). Such structural resolution may ultimately be used for high-throughput screening of IDR structural transformation(s) upon binding of ligands such as drug candidates.

## Introduction

The concept of proteins containing regions devoid of secondary and tertiary/globular structural arrangement(s) became evident in the 1970s resulting from the inability to obtain structural information with traditional measurement techniques such as X-ray crystallography and NMR spectroscopy.^1,2^ These proteins became known as intrinsically disordered proteins (IDPs) where regions lacking structure were called intrinsically disordered regions (IDRs).^3,4^ Although early studies related protein function to specific IDRs,^5^ only over the next few decades did the broad functional repertoire of IDPs emerge. Their functional significance is evidenced by their widespread observance in various proteomes where early genomic analysis suggested that the occurrence of IDPs/IDRs could be as high as ∼37%, ∼33%, and ∼51% within the Archaea, Bacteria, and Eukarya domains, respectively.^6,7^

Over the years, different classification techniques have been utilized to categorize such IDP/IDR function. In 2002, exhaustive literature searches resulted in assignments into one of twenty-eight different areas ranging from protein–protein binding to DNA bending.^7^ More recently, a categorization of six primary functions of IDRs was established to include: (1) entropic chains (remaining unstructured for function), (2) display sites (e.g., post-translational modification recognition), (3) chaperones (for RNA and protein folding), (4) effectors (e.g., protein activity modification), (5) assemblers (e.g., promoting high-order protein complex formation), and (6) scavengers.^8^

The functions of many IDP/IDR species are achieved via disordered-to-ordered structural transformations induced by molecular binding events associated with polypeptide motifs known as molecular recognition features (MoRF). Such regions can be further classified according to sequence bias (propensity to form 2° structural elements). These include those forming alpha helical (α-), β sheet (β-), and irregular (inflexible regions, *i*-) MoRF elements.^8^ IDPs/IDRs are implicated in a variety of human maladies including viral infection as well as disease progression for conditions ranging from cardiovascular to neurodegenerative disease.^9−13^

Although powerful techniques such as X-ray crystallography, (nuclear magnetic resonance) NMR spectroscopy, circular dichroism (CD) spectroscopy, protease digestion, and radius determination have been used to characterize IDP/IDR structure in the past, the various limitations associated with each technique have long been known.^14^ More recently, other techniques such as single-molecule fluorescence spectroscopy,^15^ atomic force microscopy (AFM),^16^ small-angle X-ray scattering (SAXS),^17^ and small angle neutron scattering (SANS)^18^ have been demonstrated as tools in the characterization of IDP/IDR structure and dynamics.

Mass spectrometry (MS)-based techniques for the study of IDPs have proliferated in recent years.^19^ In particular, MS footprinting techniques have been utilized to characterize IDPs. One approach, hydroxyl radical protein footprinting (HRPF) was shown to detail changes in protein modification as a function of Aβ1–42 aggregation.^20^ The use of HRFP for characterizing IDPs is especially intriguing considering the recent extension of the labeling approach to in-cell analysis.^21^ In addition to HRFP, hydrogen–deuterium exchange (HDX) labeling combined with MS has found increased usage for characterizing IDPs.^22−25^ However, each of the above-listed techniques lack some or all of the following capabilities including: (1) accurate determination of the degree of conformational flexibility, (2) resolving varying degrees of IDR bias, (3) utilizing small amounts of sample, and (4) allowing for high-throughput characterization of IDP/IDR structural flexibility and bias.

In recent years, microdroplets have been used as a reaction platform, allowing for unprecedented detail in characterization by MS.^26−28^ Zare and co-workers performed in-droplet HDX experiments to obtain rate constants for individual hydrogens.^29^ Building on this platform, we present in-droplet HDX-MS experiments for peptides exhibiting a wide range of structure and conformational flexibility. The studies are made possible via a new ionization source called vibrating sharp-edge spray ionization (VSSI)^30,31^ which provides high ionization efficiency^32,33^ while permitting facile separation of analyte and D_2_O reagent droplet plume production.^34−36^ These experiments also take inspiration from timely studies exploiting in-source HDX for distinguishing compound structures.^37−39^ Notable that, recent studies have shown that a dual emitter VSSI configuration such as that shown in Figure S1 in the Supporting Information can be used to perform in-droplet HDX to provide information about different protein and DNA conformations and their stabilities.^40−42^

Here molecular dynamics (MD) simulations are used with in-droplet HDX experiments to develop a model for relating HDX reactivity to peptide conformational flexibility. As such, this work also follows seminal studies that have demonstrated the power in coupling HDX-MS data with MD simulations for elucidating conformer-dependent reaction behavior.^43−45^ Here, this combination lays the groundwork for future rapid prediction of IDP/IDR structural flexibility and bias. A IDR structural bias is defined as the propensity to form 2° structural elements such as α-helix, β-sheet, and β-turn. With the increasing prevalence of IDPs/IDRs as druggable targets,^46−48^ this approach has the potential to offer a low-cost, high-efficiency screening strategy in the hit-to-lead and lead optimization steps of the drug development process.

## Results

### Accounting for Side Chain Exchange Using Amino Acid Standards

The total number of exchangeable hydrogens for any given peptide is the sum of those on a side chain, N- and C-terminal heteroatoms as well as backbone amide hydrogens. Because the rates of exchange for backbone and side chain sites are significantly different in bulk solutions,^49^ their relative contributions to in-droplet HDX must be accounted for separately. For the peptides studied here, side chain hydrogens can be represented primarily with those from (1) lysine (K, primary amine), (2) serine (S, primary alcohol), and (3) arginine (R, guanidino) amino acid residues. To estimate the relative contributions of side chain sites, experiments were conducted in which K, S, alanine (A), and R residues were characterized separately. Figure S2 in the Supporting Information shows a representative mass spectrum for K demonstrating the mass-to-charge ratio (*m*/*z*) shift for [M + H]^+^ ions observed upon incorporation of deuterium. The associated shift in mass, computed by the difference in the weighted average of the isotopic distributions before and after D_2_O exposure, was ∼5.16 or ∼86% of the total number of exchangeable hydrogens (*n* = 6) in the ions.

To evaluate the HDX reactivity of A, S, and R residues relative to K, multiple experiments were carried out over a series of D_2_O exposures (i.e., different internal standard exchange levels). Linear regression fitting of these data shows that A, S, and R have independent relationships (i.e., different slopes (*m*) for each best-fit line) with K (Figure S3 and Table S1). This suggests that independent intrinsic rates (*k*_int_) govern deuterium incorporation for the various exchangeable sites even over this brief reaction time scale (estimated to be ∼100 to 200 μs); the percentage of exchanged hydrogens for A, S, and R residues are ∼84%, ∼ 81%, and ∼54%, respectively. Using these data sets, it is possible to compute the propensity for deuterium exchange exhibited by each respective side chain and terminal site (Table S2) for exchange values that have been scaled to 75% deuterium incorporation for K residues. The mathematical formulation is described by the description of nonnegative linear regression as well as an analytical solution cross check using eqs S1, S2, and S3 (Table S2 footnote f).

### Deriving Backbone Exchange Levels

To demonstrate the potential of in-droplet HDX as a technique for structure determination, experiments were performed for four peptides that represent a broad range of secondary structure and backbone amide *k*_int_ rates as well as a canonical peptide associated with a neurodegenerative disorder. The variable *k*_int_ rates are represented by peptides containing the additional amino acid residues alanine (A), serine (S), aspartic acid (D), isoleucine (I), proline (P), glycine (G), phenylalanine (F), methionine (M), threonine (T), leucine (L), and glutamic acid (E). Briefly, N-terminal acetylated (Ac) Polyalanine (PA) and polyserine (PS) having sequences of Ac-PAAAAKAAAAKAAAAKAAAAK and Ac-PSSSSKSSSSKSSSSKSSSSK, respectively, were used to provide diverse structures for study. These peptides have been shown to form either highly helical (PA) or disordered (PS) structures in solution,^50−53^ The other peptides have additional amino acid residues including the design peptide KDD having the sequence KKDDDDDIIKIIK. This peptide is relatively unstructured and engineered to have fast- (D residues) and slow-exchanging (I residues) backbone amide regions.^54−56^ Bradykinin (BK) is a nonapeptide with the sequence RPPGFSPFR. BK is largely unstructured but possesses a distinct β-turn at the C-terminus.^57^ Finally, Nt17 is the N-terminal portion of Exon 1 from the Huntingtin protein having the sequence MATLEKLMKAFESLKSF. Nt17 forms an amphipathic helix with hydrophilic and hydrophobic regions upon interaction with a binding partner (e.g., peptide or lipid bilayer).^58^ Overall, the variability in disorder and backbone amide *k*_int_ for these peptides (albeit limited in sample numbers) is sufficient for first approximation testing of the experimental and computational approach.

**Figure 1:**
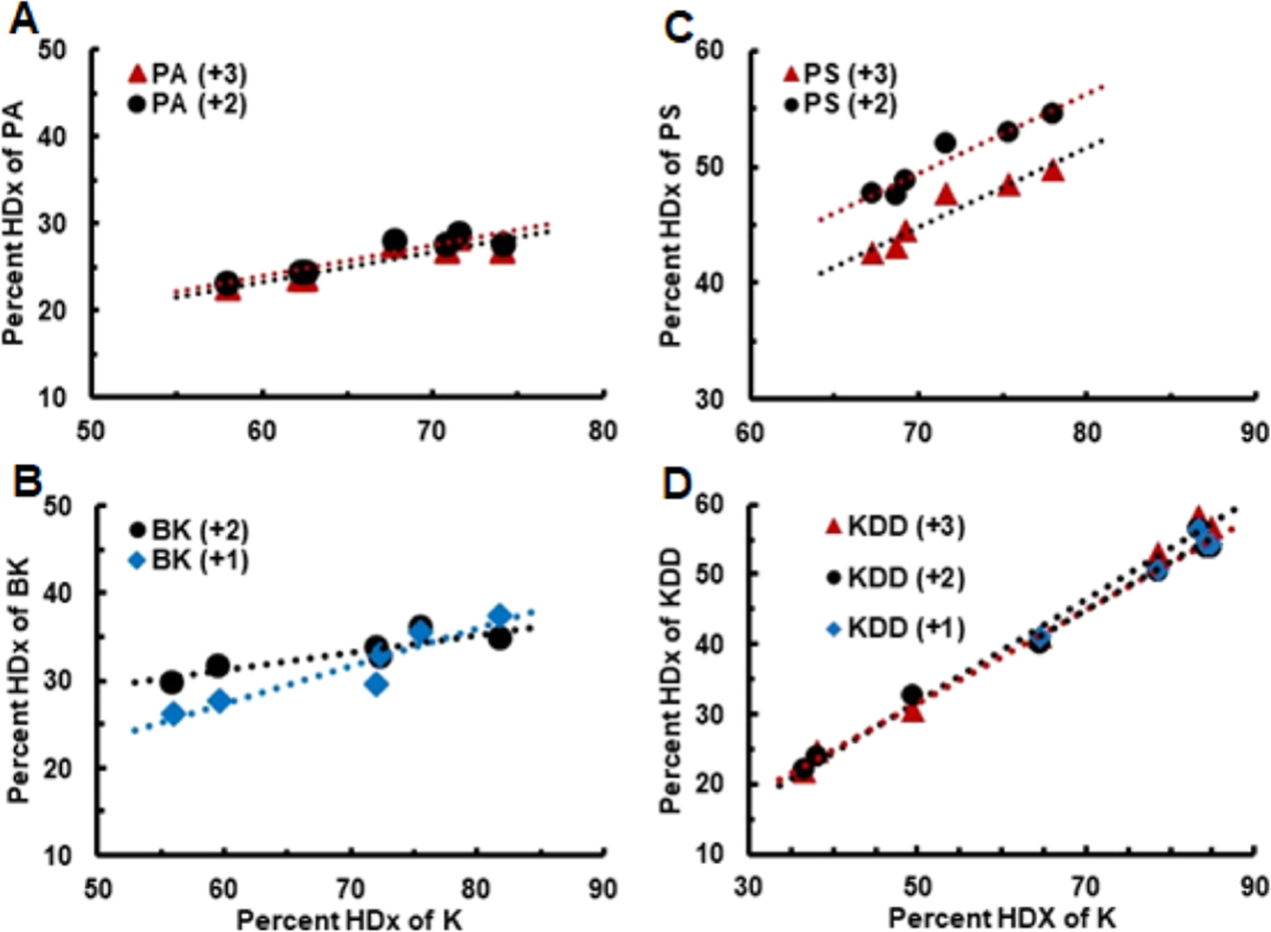
HDX behavior of the different model peptides. Scatter plots of analyte, PA (Panel A), PS (Panel C), BK (Panel B), and KDD (Panel D). Inset legends provide colors and symbols for charge states. Best-fit lines (dotted lines) are shown and were obtained with the LINEST function in Excel. See Table S1 in the Supporting Information section.

Figure S4 shows representative HDX data for each peptide upon exposure of the corresponding analyte droplets with the D_2_O droplets. As with the individual amino acid residues, the HDX behavior of each peptide can be referenced with respect to that of [M + H]^+^ K ions as shown in Figure 1. [M + H]^+^, [M + 2H]^2+^, and/or [M + 3H]^3+^ ions correspond to major features within the analyte mass spectra, allowing this characterization of peptide dynamics with at least two different charge states. A comparison of the slopes (*m*) obtained from HDX data (Figure 1) reveals peptide-specific behavior, ranging from an average of ∼0.31 and 0.34 (BK and PA) to ∼0.68 and 0.72 (PS and KDD) (Table S3). Using the internal standard K, it is possible to scale the deuterium incorporation level of each peptide to represent similar exposure conditions (e.g., D_2_O/H_2_O ratios leading to the same internal standard exchange of 75%). Average exchange levels obtained from this scaling process are presented in Table 1 for each peptide. For a detailed description of average exchange value, see the HDX Calculation section in the Supporting Information section.

**Table 1:** Percent Deuterium Incorporation for Side Chain and Backbone Sites

peptides	HDX (Unc.)^a^	exchangeable sites^b^	side chain contribution^c^	% backbone (% BB) HDX^d^ (Unc.)
PA	9.1 (0.3)	9/20	8.79	1.5 (1.3)
Nt17	12.1 (0.2)	14/16	11.09	6.1 (1.4)
BK	6.0 (0.1)	12/5	5.07	17.6 (1.3)
KDD	14.7 (0.1)	14/12	13.37	10.8 (0.5)
PS	24.0 (0.5)	25/20	17.79	31.0 (3.0)

a Total deuterium uptake scaled to 75% deuterium incorporation by the internal standard K (Figure 1). The uncertainty in deuterium uptake given parenthetically represents the propagated error (*m* and *b* from the best fit lines in Figure 1) followed by the propagation of error for the weighted charge states (see HDX Calculation in the Supporting Information section). The number of mass spectral replicates and individual measurements for each peptide are provided in the Methods section.

b Numbers of side chain/backbone exchange sites on each neutral peptide.

c Side chain deuterium incorporation determined using the HDX propensity values from Table S2.

d % Backbone deuterium incorporation as computed from the difference of total and side-chain deuterium incorporation expressed as a percentage of total backbone hydrogens. Uncertainties given parenthetically represents propagation of the error from the HDX (first column) as shown in the HDX Calculation section of the Supporting Information section.

Having characterized side chain exchange contributions adequately, it is possible to determine relative levels of peptide backbone amide exchange.^34^ Using the HDX propensity factors for the side chain sites (Table S2), we determine the contribution by side chain sites to the total deuterium incorporation for each respective peptide as described previously.^34^ Subtracting side chain contributions from the total deuterium uptake produces the backbone amide exchange levels. For comparison across peptides, it is useful to express these values as the percentage of the total backbone amide hydrogens (% BB). % BB exchange values for each peptide are presented in Table 1. For a detailed description of the calculation of % BB exchange, see the HDX Calculation section in the Supporting Information. Marked differences between the % BB exchange levels exist for this series of peptides, ranging from ∼1.5% (PA) to ∼31% (PS). These distinct values are here proposed to stem from differences in preferred structural conformations, but further understanding of this will require more definitive structural characterization, which is pursued via molecular dynamics (MD) simulations.

**Figure 2:**
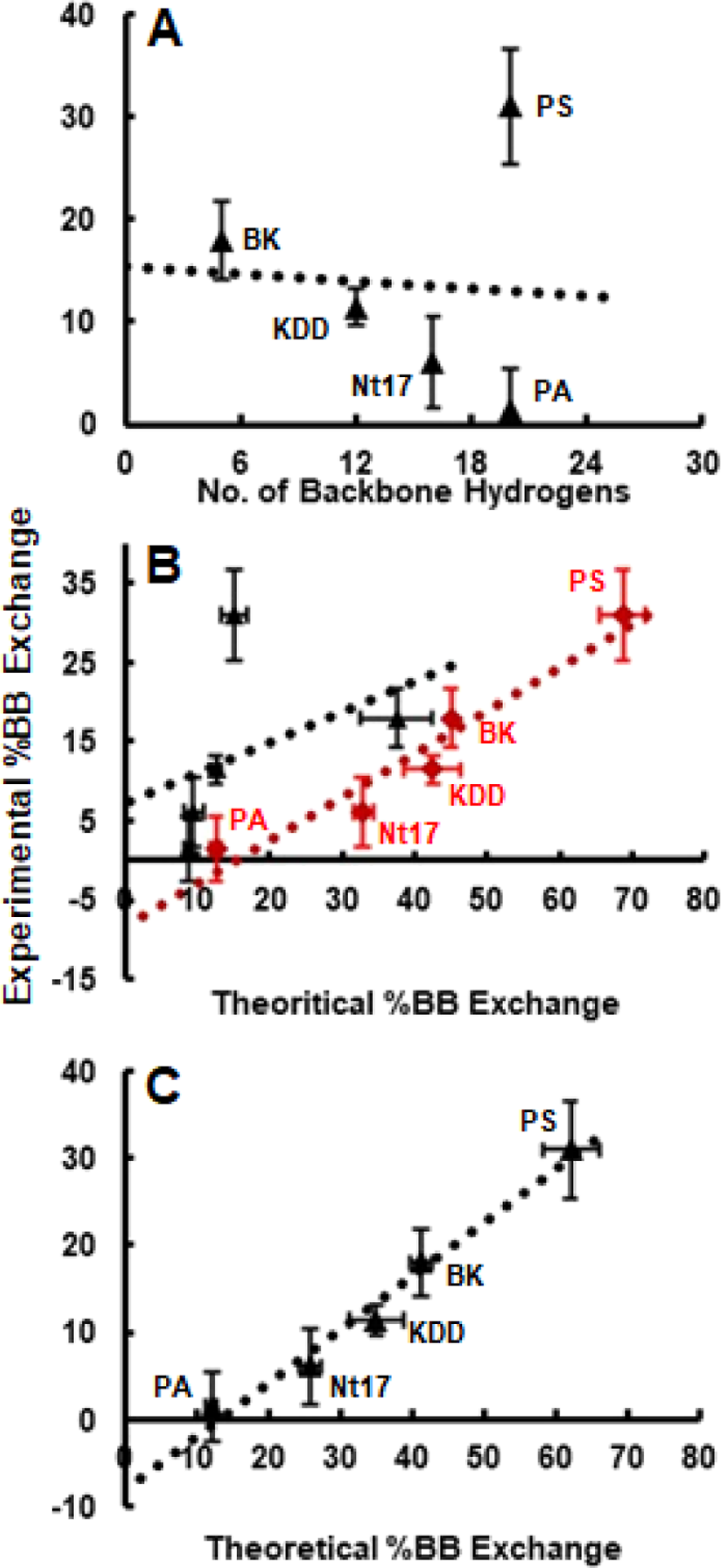
Relationship between theoretical and experimental exchange values for the various models described in the text. Panel A shows the experimental % BB exchange as a function of total backbone exchangeable sites for all peptides. Panel B shows the experimental % BB exchange as a function of the theoretical % BB exchange for the *M*_intra_ (red symbols) and *M*_inter_ (black symbols) models for all peptides. Panel C shows the relationship between experimental % BB exchange and theoretical % BB exchange for the *M*_CMB_ model. Dotted lines show the best-fit lines obtained from the LINEST function (see Table 2). Individual peptides are labeled in each panel. *y*-axis error bars (measurement uncertainties) are obtained by propagation of error and *x*-axis error bars represent standard error of the meant. These calculations are provided in the Error Calculation section of the Supporting Information.

It is instructive to consider the relationship of the experimental % BB exchange levels with the total number of backbone amide hydrogens for each peptide. Figure 2A shows this comparison. A linear least-squares fit analysis of these data (see the backbone exchange model—*M*_BB_—in Table 2) reveals essentially no correlation (*R*^2^ ∼ 0.004) between the two factors. The poor correlation suggests that different peptide solution structures lead to varying protection of backbone amide hydrogens. An exploration of primary, secondary, and tertiary structural differences leading to varied HDX protection is the subject of the MD simulations work described below.

**Table 2:** Regression Analysis Statistics for % BB Exchange Estimations by Different Models

model	*m*^a^	*b*^b^	*R*^2^^c^	SS_res^d^	% error^e^
*M*_BB_	–0.12 (1.05)	15 (16)	0.004	527	200 (150)
*M*_inter_	0.4 (0.5)	7 (10)	0.16	443	160 (100)
*M*_intra_	0.54 (0.09)	–8 (4)	0.93	39	60 (40)
*M*_CMB_	0.61 (0.06)	–8 (2)	0.98	13	40 (30)

a Slope obtained from the LINEST function in Excel. The error is given parenthetically as defined in the Error Calculation section of the Supporting Information section.

b *y*-intercept obtained from the LINEST function in Excel. The error is given parenthetically as defined in the Error Calculation section of the Supporting Information section.

c Square of the correlation coefficient obtained from the LINEST function in Excel.

d Residual sum of squares from the LINEST function in Excel.

e Average percent error obtained using *m* and *b* values to compute theoretical exchange when compared to the experimental exchange. The error is given parenthetically and represents standard error of the mean (see Error Calculation section).

### MD Simulations to Calculate Protection Factors and Relate Them to Peptide HDX Levels

MD simulations have been utilized in the past to estimate HDX reactivity. In particular, solvent-accessible surface area (SASA) measurements in combination with the tracking of hydrogen bonding (HB) of amide hydrogens are shown to be reliable for quantitatively differentiating protein conformation populations (i.e., open and closed states).^59^ In the present work, two HDX models (i.e., *M*_intra_ and *M*_inter_) obtained from MD simulations data were evaluated for their ability to provide structural information. Both models assume that HDX can accurately characterize open (*c*_o_) and closed (*c*_c_) conformational states.^60^ In brief, both models utilize SASA while inter- and intramolecular HB are included in the *M*_inter_ and *M*_intra_ models, respectively. Notably, the *M*_inter_ model described below is very similar to that reported in the literature for protein studies.^59^ Here it is tested for highly dynamic peptides.

To combine HDX modeling with MD simulations, the determination of exchange events must be based on the relative degree of *c*_o_ (i.e., unprotected/unfolded) and *c*_c_ (i.e., protected/folded) conformational states. This relationship can be expressed as follows^60^
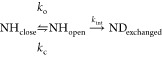
1where NH represents an individual exchange site (hydrogen attached to a heteroatom), *k*_o_ is the closed-to-open state rate constant, *k*_c_ is the open-to-closed state rate constant and *k*_int_ is the exchange reaction rate constant. One hypothesis is that the ratio of *c*_c_ to *c*_o_ states determines the relative resistance to exchange. This can be represented by the protection factor (PF—simply expressed as *k*_c_/*k*_o_) experienced by each site. Utilizing the relationship in eq 1, under steady state conditions the overall rate of exchange would be *k*_obs_ = *k*_o_*k*_int_/(*k*_o_ + *k*_c_ + *k*_int_).^61^ In the case of a small peptide, as a first approximation, it can be argued that *k*_o_ > *k*_int_ and *k*_c_ > *k*_int_ and the *k*_obs_ can be approximated by the PF and *k*_int_ as shown in eq 2.^62^
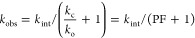
2

The PF from eq 2 can be related to results from MD simulations using
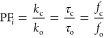
3where *i* represents the *i*th exchange site, and τ_c_ and τ_o_, and *f*_c_ and *f*_o_ represent resident time and fractional population in the *c*_c_ and *c*_o_ states, respectively. Thus, for the amide hydrogen of the *i*th residue, PF_*i*_ can be estimated directly from the MD simulations using
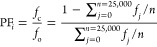
4

In eq 4, *f*_*j*_/*n* represents the fraction of *c*_*o*_ states (from *n* = 2.5 × 10^4^ structures across the MD trajectory) for the *i*th residue. Following from
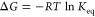
5where *ΔG* and *K*_eq_ (where *K*_eq_ = *f*_o_/*f*_c_) are referenced to the HDX reaction occurring for each site *i*, effectively the contribution to overall peptide exchange can be represented by totaling the number of times that the ln(PF_*i*_) values are less than 0. This summation represents the theoretical amide backbone hydrogen exchange for each peptide.^62^

Notably, for model development here, the determination of *f*_o_ (and relatedly *f*_c_—see eq 4) for each site in a MD structure is similar to that reported previously for estimating HDX reactivity.^59^ In the *M*_inter_ model evaluated here, the *c*_o_ state exists if and only if SASA_*i*_ of the amide backbone hydrogen (NH_*i*_) is greater than or equal to 8.3 Å^2^ and there are at least two water molecules within 3.3 Å and a 30° angle threshold representing strong HB criteria; otherwise, the exchange site is assigned the *c*_c_ state. In the *M*_intra_ model, the *c*_c_ state is defined as SASA_*i*_ < 8.3 *Å* and/or at least one intramolecular hydrogen bond is being formed by the amide hydrogen (NH_*i*_)*;* otherwise, the *c*_*o*_ state is assigned.

**Figure 3:**
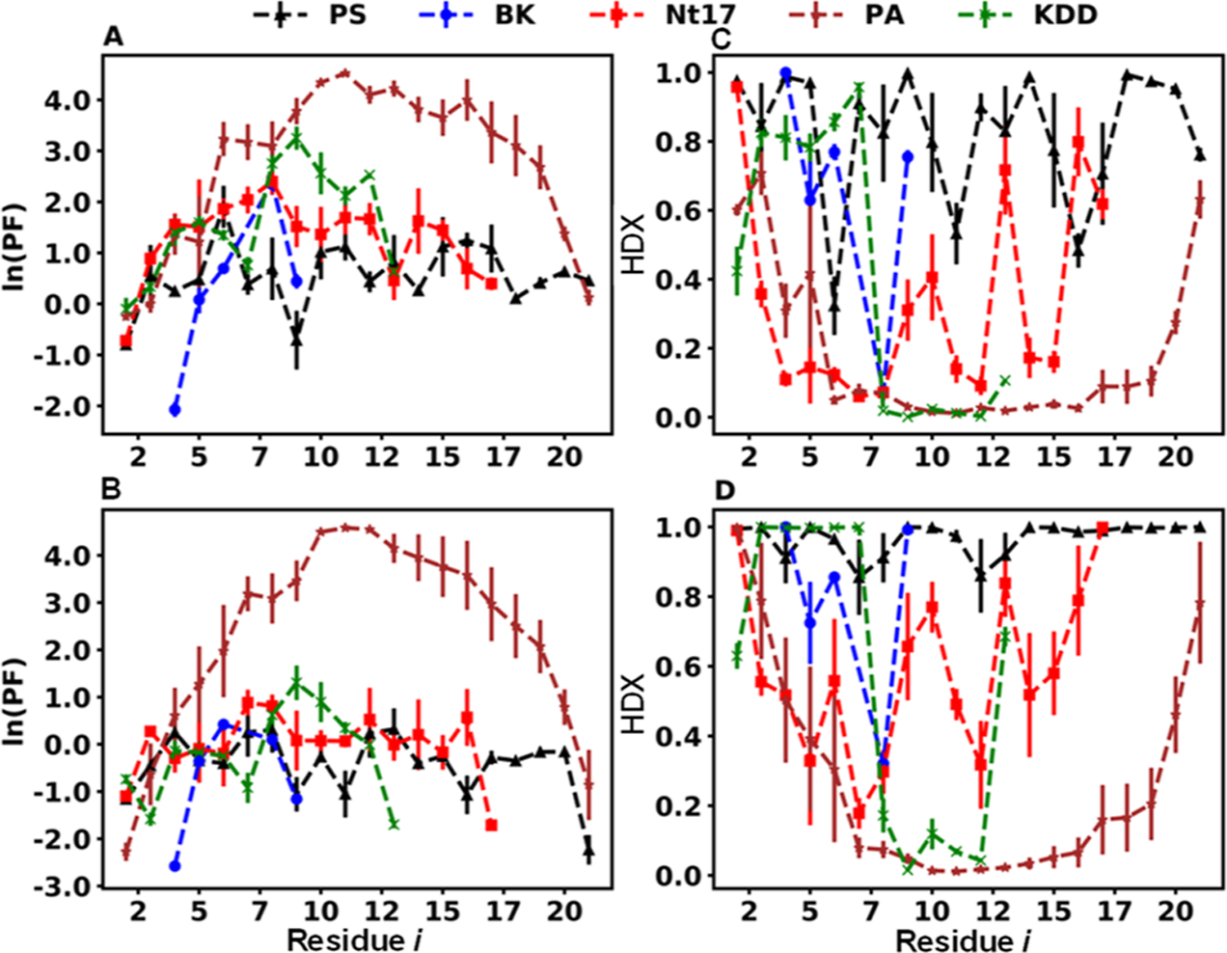
HDX analysis as described by PF_*i*_ for the model peptides. For *M*_inter_ and *M*_intra_ models, panels A and B, respectively, show the ln(PF_*i*_) data for PS, BK, Nt17, PA, and KDD. Panels C and D (respective models) show the per-residue HDX contributions for the same peptides using the *M*_CMB_ approach with the inter- (panel C) and intramolecular (panel D), HB treatment, respectively. To obtain these HDX contribution values, eq 6 was used (see text for details). Error bars represent the standard error of the mean from multiple (*n* ≥ 3) replicates (see Error Calculation section in the Supporting Information).

Figure 3A,B show example ln(PF_*i*_) values for peptide backbone amide sites upon application of the *M*_inter_ and *M*_intra_ HB treatment, respectively, to the MD trajectories obtained here. A comparison of Figure 3A,B shows that generally the PF_*i*_ values decrease for all peptides with application of the *M*_intra_ treatment suggesting greater hypothetical backbone exchange. This change is most pronounced for the peptides other than PA as evidenced by the decreased overlap in ln(PF_*i*_) values with the PA peptide especially in C-terminal regions (Figure 3B versus 3A). Translation of ln(PF_*i*_) values into hypothetical exchange values is achieved as mentioned above where any negative value would represent exchange and positive values would not. The per-peptide sum of these values can then be used to calculate a theoretical % BB exchange for each peptide using the *M*_inter_ (Figure 3A) and *M*_intra_ (Figure 3B) models. These values can be compared with the experimental % BB exchange values. Figure 2B shows these relationships for the *M*_inter_ and *M*_intra_ models. Briefly, the correlation obtained for the latter is substantially more significant than that obtained for the former (essentially nonexistent). This is clearly indicated by the linear least-squares best-fit statistics presented in Table 2. Of note, the squared sum of the residuals (SS_res) and the average %Error for this relationship decrease by ∼10 and ∼3 fold (Table 2), respectively, with the use of the *M*_intra_ model over the *M*_inter_ model.

### Combined Empirical and Physics-Based Model Provides Significant Improvement in Agreement of Experimental and Theoretical Exchange Values

Although the *M*_intra_ model shows a modest correlation with the experiment, it does not capture well the exchange levels reported for those peptides exchanging fewer hydrogens (see results for PA, KDD and Nt17 in Figure 2B). Additionally, the *R*^2^, SS_res, and % error values (0.93, 39, and 60 Table 2) associated with this model suggest limited analytical utility. It is thus necessary to extract semiquantitative information for improved model comparison. That is, although PF_*i*_ has been used in the past to estimate the sign of Δ*G* (eq 5) for HDX reactions of given sites, the digital assignment to exchange event may lead to a over-representation or uncertainty of estimated in-droplet exchange (generally greater *x*-axis values for *M*_intra_ in Figure 2B).

One of the first attempts to develop a model to account for backbone amide exchange used NMR to characterize residue-specific HDX *k*_int_ as a function of temperature and pH.^63^*k*_int_ is based on the primary sequence, which captures electronic and inductive effects of neighboring amino acid residues and is defined in ref (63) for different amino acid residues. With values for *k*_int_, it is possible to obtain estimated HDX values for backbone amides of the different peptides using
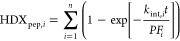
6

In eq 6, *t* represents reaction time. It is noted here, that for the purposes of obtaining a strong correlation between experimental and theoretical % BB exchange, *t* values are arbitrary. For example, using *t* = 100 μs and an accelerated *k*_int_ as obtained from earlier experiments^34^ provides HDX_pep,*i*_ values that are identical to that on a 1 s time scale in bulk solution. That said, eq 6 forms the basis of the last model discussed here which is called the combined model (*M*_CMB_). Figure 3C,D show the per-residue amide HDX values (*n* total) calculated using eq 6 with the *M*_inter_ and *M*_intra_ HB treatment, respectively. From these figures, it is clear that when PF_*i*_ is represented by the *M*_inter_ HB treatment, less theoretical % BB exchange is computed for all peptides. That is, the average per-site HDX value is decreased in Figure 3C relative to that in Figure 3D. Consider the values for the PS peptide. For residues 12 to 20, 6 of the 9 amide sites have an HDX contribution value below 0.9 when the *M*_inter_ (Figure 3C) method is employed whereas only 1 of the same 9 amide sites exhibits a value below 0.9 with the *M*_intra_ (Figure 3D) HB treatment.

Because the *M*_CMB_ model generally shows a greater theoretical % BB exchange for peptides when using the *M*_intra_ HB treatment (Figure 2B), it is instructive to examine the relationship between the experimental and theoretical % BB exchange for *M*_CMB_. Figure 2C shows the correlation between these values. The *M*_CMB_ model is superior to the previous models in every respect, providing a statistically significant (*R*^2^ ∼ 0.98, Table 2) relationship. A noticeable correlation improvement is also evidenced with gains of ∼3 and ∼1.33 fold for SS_res and %Error compared with the *M*_intra_ model (Table 2). Finally, the x- and *y*-axis ranges show improved similarity by ∼1.13 fold indicating a smaller overestimation of % BB exchange by *M*_CMB_.

### Structural Insights Gained by Correlating HDX-MS and MD Simulations Results

It is instructive to infer the structural origins of the HDX behavior of the model peptides using the MD simulations data. For example, BK is a 9-residue peptide with a high degree of disorder despite which BK exhibits significantly lower experimental backbone reactivity than the unstructured PS peptide (Figure 2C). Molecular flexibility could be one factor explaining the apparent discrepancy. Studies of the NMR order parameter demonstrated that primary chain fluctuation depends on the side chain constituents; larger side chains resulted in lower backbone fluctuation.^64^ Therefore, more limited backbone fluctuation may account for a portion of the limited HDX behavior of BK and this may be captured in the MD simulations results.

From the MD simulations, secondary structure evolution can be evaluated over the 500 ns trajectory. Figure S5 depicts the PA secondary structure changes over this time frame. The middle 16 residues form an α-helix conformation while the other residues form extended helix or coil conformations. This suggests that the low deuterium incorporation for PA results from intramolecular HB. For BK (Figure S6), although no strong intramolecular HB is evident, the peptide mainly remains in a single conformational state comprised of random coil and turn rendering it conformationally inflexible. The peptide KDD shows increased flexibility with mostly coil interspersed by a very transient helix, extended helix, and turn formation occurring at different residues (Figure S7). The PS peptide exhibits the greatest degree of flexibility (Figure S8); fluctuations occur rapidly between coil and turn conformations.

Backbone deviation can also be examined to understand conformational flexibility. For PS, because it can sample essentially any structural form, normalized density plots of the backbone RMSD show a broad area ranging from 7 to 18 Å demonstrating higher frequency state-to-state conversions (Figure S9). KDD is a designed peptide containing both fast- (N-terminal portion) and slow-exchanging (C-terminal portion) regions.^54−56^ Deuterium incorporation for KDD falls between that of BK and PA (closer to the former). Figure S7 suggests that the first 7 residues undergo greater structural fluctuation than the remaining residues. Comparatively, its backbone deviation (Figure S9) is comprised of two broad features ranging from 3 to 6 Å and 7 to 10 Å near the range exhibited by PS. This may suggest how *M*_intra_ obtains a relatively high theoretical % BB exchange level (near that of BK) for this peptide (Figure 2B) yet the addition of *k*_int_ reduces the theoretical % BB exchange (Figure 2C) more than that of BK. Finally, the backbone deviations for BK and PA (Figure S9) are narrower in range indicating reduced flexibility. The greater structural rigidity of the PA peptide is clearly evident in the RMSD distribution. Additionally, conformational states comprising two different structure regions/types for BK (Figure S9) show greater flexibility compared to PA. These data for PS show the greatest scatter (no significant accumulation in any area) suggesting the greatest flexibility.

The molecular asphericity (δ) for the different peptides as monitored by the radius of gyration (*R*_g_) over time may be another indication of flexibility. Figure S10 shows this relationship representing the frequency of occurrence (color map) over separate MD trajectories. These data show a clear increase in structural flexibility going from PA to BK to KDD to PS as more states become populated (larger spread shown in Figure S10).

A residue contact map reveals the propensity of each peptide residue to form α-helix structure. Contact maps for each peptide are shown in Figure S11. For PA, frequent contact between neighboring residues is associated with helix formation throughout the trajectory run. For BK, strong interaction occurs between residues 4 and 2 across one trajectory run and 5 and 8 across two other trajectories. This suggests some conformational rigidity but not as much as PA. Heat maps for KDD and PS (Figure S11) show increasing conformational flexibility because of the decreased prevalence of singular inter-residue interactions.

A short note on the BK structural inflexibility is that it occurs because of the “locking in” of structure in the presence of three proline (P) backbone-constraining residues and large R residues (see sequence above). In that regard, BK and PA show similar trends in the residue contact map (Figure S11) revealing a sharp feature occurring between 4 and 5 Å. As BK experiences a greater overestimation by *M*_CMB_, it may again be suggested that in the future *M*_CMB_ need be modified to increase the influence of different types of conformer rigidity.

### Structure and Dynamics for a Disease-Relevant Peptide

The % BB exchange exhibited by Nt17 falls between that recorded for PA and BK and KDD (Figure 2C). Because Nt17 is shown to be unstructured at the experimental concentrations employed, it might be expected that, with such experimental flexibility, experimental % BB exchange values should be closer to PS (the largest of all peptides). It is intriguing to consider that the presence of structural inflexibility and/or secondary structure of some form may thus hinder HDX by Nt17.

**Figure 4:**
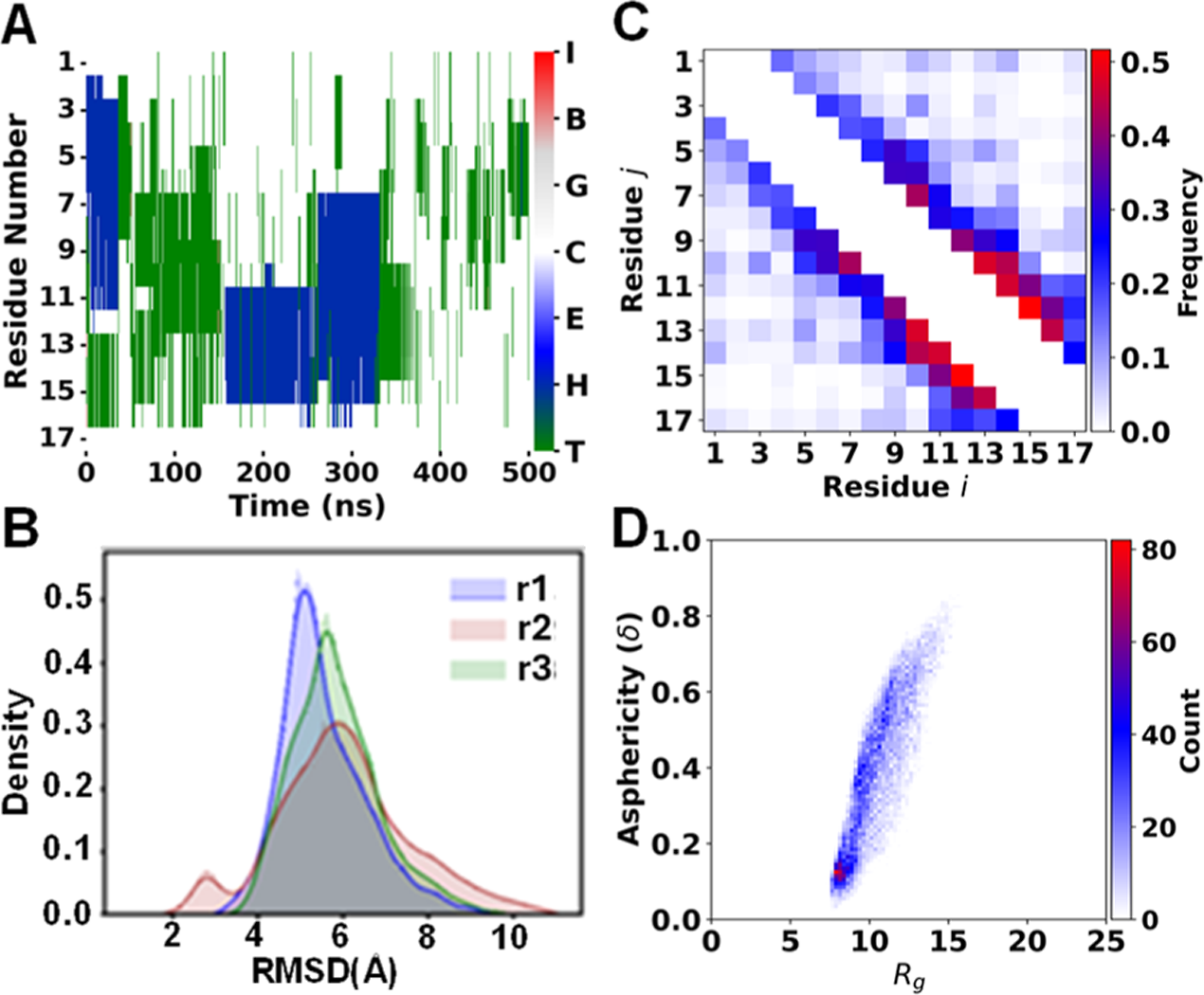
Secondary structure analysis of Nt17. Structural evolution plots including: Panel A showing helical content, Panel C showing a 2D histogram of residue (*i*) vs residue (*j*) contact distribution, Panel B showing a density plot of backbone root-mean-square deviation (RMSD), and Panel D showing the radius of gyration (*R*_g_) vs Asphericity (δ). In Panel C, the Frequency (unitless) color bar represents the degree of an event (*i* and *j* coming within HB contact distance). The Density (unitless) in Panel C refers to the fraction of conformers having the RMSD value and r1, r2, r3 refer to 3 separate MD trajectories. For results from replicate MD simulations, see Figures S12–S14.

Figure 4 shows the residue helical propensity across the MD trajectory for Nt17. Residues 3 to 13 can rapidly alternate between helix, extended helix and coil structures. Overall, the peptide flexibility falls between that of PA and BK and KDD which is consistent with this peptide exhibiting an experimental % BB exchange level that is closer to PA than unstructured PS. Notably, such structural transition can be argued to be important for its ability to form and stabilize helical conformation upon associating with a binding partner (lipid or protein) in the aggregation process associated with Huntington’s Disease.^58^ A strong α-MoRF property for Nt17 is supported by the residue contact map shown in Figure 4. Here, associations (3 to 4 residues apart) are observed for residues 4 to 14 across the MD trajectory. Backbone RMSD (Figure 4) evaluation confirms that Nt17 flexibility falls between that of PA and BK and KDD (Figure S9) suggesting increased transient helix formation. Finally, the δ versus *R*_g_ plot (Figure 4) shows some scatter indicating conformational flexibility; however, structural restraint is observed in the concentration of some forms at the bottom of the distribution (again pointing to helical propensity).

## Discussion

### Evidence for a Solution Exchange Process

The utility of *M*_CMB_ for ascertaining polypeptide structure, structural-flexibility, and structural-bias requires that gas-phase HDX not substantially alter ion deuterium content. Prior studies provide evidence supporting a primary solution exchange process.^34,35^ Briefly, separate experiments have shown that protonated and potassiated glycan ions produced in the same experiment exhibit the same HDX behavior. The former ions are capable of exchanging in the gas phase but the latter are not via the relay mechanism.^65^ The similarity in exchange suggests reactions occur primarily in solution under the same exposure conditions (D_2_O/H_2_O ratio). Separate experiments show that the [M + H]^+^ and [M + 2H]^2+^ BK ions undergo similar levels of exchange as also observed in these studies (Figure 1). [M + H]^+^ BK ions essentially do not undergo gas-phase exchange while the [M + 2H]^2+^ ions readily undergo HDX.^66,67^ This again suggests similar reaction conditions which for the [M + H]^1+^ ions must be almost exclusively in solution. Additionally, recent studies of the protein ubiquitin suggest a range of HDX reactivities that is not typically accessed for gas-phase HDX.^34^ Finally, that the *M*_CMB_ model, which relies heavily on solution modeling, well accounts for the HDX behavior of the peptides of such diverse structural flexibility further supports solution-phase reactions. That said, some gas-phase HDX may occur and may limit the overall utility of *M*_CMB_.

### IDP/IDR Structure Determination

The elucidation of IDP structure is paramount considering its prevalence within eukaryotes and association with disease processes. To utilize in-droplet HDX for IDR structural flexibility, the model would need to be extended to larger proteins. First, the HDX propensity factors (Table S2) would have to be determined for all side-chain hydrogens for the solution conditions (ionic strength and pH) employed. Once determined, MD simulations could be used as described herein to obtain estimates of deuterium incorporation in structured and unstructured regions of the protein. Next, the MD trajectories would be examined to verify that the HDX model indeed captures the exchange within IDRs. A future goal would be to link exchange levels to MoRF type and structure bias. Establishing this, it would be possible to ascertain structural changes occurring upon specific events such as protein–ligand binding. This may be especially suited to drug screening and drug design.

### High-Throughput Screening

The VSSI approach offers several advantages for high-throughput screening with in-droplet HDX. First, VSSI often provides superior ionization efficiency compared with standard ESI and nESI.^32,33^ Second, VSSI has been shown to better preserve the solution structures of some highly flexible species when operated in a voltage-free fashion.^42^ Additionally, the VSSI approach allows the decoupling of droplet production from the Coulombically driven process of ESI. Thus, one can access a greater diversity of droplet sizes and charges from single emitter tips to vary and/or tailor HDX reagent levels within coalesced droplets. Finally, analyte emitter tips can be highly parallelized to allow high-throughput screening capabilities.

One type of enabled screening envisioned is the examination of drug libraries with IDPs. Here, consider for a 96-well plate process, a similarly sized array of VSSI emitter tips could be dipped into the test wells. Then each emitter tip representing a different protein–drug interaction could be sequentially utilized for MS analysis in an automated fashion. Changes in IDR flexibility and structural bias for hundreds of systems could then be probed on a relatively short time scale. Beyond such protein–ligand interactions is the possibility to observe structural change due to other factors such as changes in the local microenvironment (e.g., pH, ionic strength, etc.).

Another variation of enabled high-throughput screening for drug development could involve analyses of designed peptide flexibility and bias for drug delivery. For example, recent work has shown that structural flexibility of Huntingtin oligomers (containing Nt17) plays a key role in lipid membrane binding.^68^ The in-droplet HDX approach may permit the high-throughput screening of many different MoRF sequences such as Nt17 (and PTMs) to determine their overall structural flexibility and bias. Then, in similar high-throughput fashion, the affinity for different membranes could be probed.

### Future Work

As described herein, one goal of developing a structure-to-reactivity curve would be to establish the ability to predict structure flexibility and bias. In such applications, only the HDX reactivity would be measured to obtain structural information. To improve HDX predictability, a much larger peptide cohort will be studied to develop separate models for similarly behaved peptides. For example, prediction models may be developed for α-MoRFs, β-MoRFs, and *i*-MoRFs using tuning of different parameters (e.g., weighting of *k*_int_ or SASA threshold values and/or treatment of side-chain exchange levels). A goal is to obtain the highest resolution for distinguishing conformer change (structural flexibility and/or bias). Particularly timely are developments in MD simulations that would benefit this work. For example, the modeling of structural behavior in nanodroplets has resulted in the prediction of ion formation processes.^69−71^ As a portion of the ions’ time is encountered in nanodroplets, it may be necessary to model HDX in the micro- and nanodroplet environment in the place of a water-box simulation. Separate studies have shown that conformational weighting of MD-generated structures is a powerful tool to better predict HDX protection.^72^ Relatedly, to help improve the filtering of structures, experimental improvements such as the coupling of in-droplet HDX-MS with a nonergodic ion fragmentation technique such as electron transfer dissociation (ETD)^73^ may be required. This would provide knowledge of the per-residue location of deuterium incorporation.^74^

With improved structural representation from the MD simulations, it can be envisioned that machine-learning/AI approaches may be applied to optimize the model(s) and obtain the best reactivity-to-structure calibration curves. For example, recently structure-based geometric deep learning has been used to predict the absorption wavelength of rhodopsins based only on their primary sequence.^75^ Such an approach employing graphical relationship rather than Euclidian distance relationship comparisons may be useful for filtering structures and/or deciphering the best usage of such factors as SASA and H-bonding for in-droplet HDX reactivity estimation.

Here a note of caution is introduced. The use of the term prediction in the context of a reactivity-to-structure calibration curve does not imply an accurate determination for each individual exchange site. If this were possible, slope and intercept values of the best-fit line for the *M*_CMB_ model (Figure 2C) would be precisely 1 and 0. From Table 2, it is observed that these values are 0.65 and 0.3, respectively. Although this model comes the closest of all models presented here to values that would represent a true representation of each exchange site, the relationship between theoretical % BB exchange and experimental % BB exchange is relative. That is, given that individual molecules within conformer ensembles are likely to display a variety of exchange patterns, it would be impossible to obtain a 1 to 1 correlation between experiment and theory for each exchange site. Thus, the structure prediction discussed hereafter refers to the potential to use a reactivity-to-structure calibration curve to predict relative amounts of secondary structural elements or the relative degree of structural bias.

To realize the full potential of reactivity-to-structure prediction would necessitate the phasing out, or diminished use, of MD simulations. That is, it is here envisioned that highly informative in-droplet HDX reactivity measurements alone can provide immediate structure information that would be useful for biophysical studies. For example, a recent study evaluated the α-MoRF pH-low insertion peptide (pHLIP) for its ability to deliver kDa-sized molecules to tumor cells.^76^ Under acidic conditions, such a peptide forms a helix at the surface of a membrane facilitating insertion. Therefore, a goal of drug discovery may be to develop peptide sequences that retain, or improve upon, α-MoRF character upon drug conjugation and/or membrane association. It is noted that the Nt17 peptide used in these studies is an α-MoRF species that associates with membranes. Remarkably its HDX signature is better approximated by the helical PA behavior than that of the unstructured PS peptide. In a sense, the relationship shown in Figure 2C may already find utility in that different pHLIP (or related peptide) mutants can be rapidly screened to observe where they fall on this calibration curve. That is, could species be found that experience % BB exchange much closer to PA than Nt17?

Extending the methodology to protein–ligand systems will also be a future goal. For example, one approach would be to determine the change in structure and structural flexibility of both interactive and allosteric sites. Developing utility for more complex systems will require foundational studies such as those presented here. To accomplish this, select (well-behaved) systems can be examined. One example could be the study of the human immunophilin FKBP-12/rapamycin complex (PDB Entry—1FKB). As the FKBP-12 protein contains multiple IDRs including regions involved in binding, in-droplet HDX may reveal changes in structure and flexibility in such regions between bound and unbound forms. Initially, such foundational studies will require extensive MD simulations work as demonstrated here. However, with ever-increasing measurements, it may be possible to incrementally remove model inputs (e.g., complete MD trajectories) to gain important structural insight.

The removal of model inputs (or indeed of MD entirely as with the pHLIP peptide example above), could eventually enable a high-throughput approach where structural insight is obtained from rapid sequential screening. High-throughput screening will require other types of experiments such as those required to obtain a sound understanding of other phenomena associated with droplet mixing. For example, droplet size and charge dependence on vibrating tip diameter, amplitude, and applied voltage will be established. Additionally, droplet shrinkage rates for those of different sizes and charges will be explored. This will result in the optimization of VSSI devices, analyte and reagent flow rates, and source geometry for the desired high-throughput experiments.

## Materials and Methods

### Reagents

For these studies, the following chemicals were purchased from Sigma-Aldrich (St. Louis, MO, USA): ammonium acetate, D_2_O, and the amino acids (A, S, K, and R). The peptides PS (Ac-PSSSSKSSSSKSSSSKSSSSK), PA (Ac-PAAAAKAAAAKAAAAKAAAAK), KDD (KDDDDDIIKIIK), and Nt17 (MATLEKLMKAFESLKSF) were purchased from Genscript (Piscataway, NJ, USA). Finally, BK (RPPGFSPFR) was purchased from Sigma-Aldrich (St. Louis, MO, USA). For amino acid propensity factor experiments, a 50 mM ammonium acetate solution containing 0.1 mg/mL of each amino acid was prepared. Analyte solutions for the peptides were prepared with a final concentration of 20 μM peptide and 100 μM internal standard (K and R) in 50 mM ammonium acetate.

### Construction of VSSI Devices

VSSI devices were fabricated as described previously.^32^ First, a 4.6 kHz piezoelectric transducer (Murata Electronics, Smyrna, GA, USA) was attached to the end of a microscope glass slide (no. 1, VWR, Radnor, PA, USA) using 5 min epoxy. Next, a short piece (5.5 cm in length, 100 μm ID × 360 μm OD) of pulled (P2000 micropipette puller, Sutter Instrument, Novato, CA, USA) glass capillary (Polymicro Technologies, Phoenix, AZ, USA) (ID ∼ 20 to 30 μm) was attached at the edge of the glass slide. A radio frequency (RF) function generator (Tektronix AFG-1062, Beaverton, OR, USA) was used to apply a sinusoidal wave (93 to 100 kHz, ∼ 80 to 100 mV_pp_) to an amplifier (Krohn-Hite 7500, Brockton, MA, USA). The output voltage (∼10 V_pp_) was applied to the transducer to produce tip vibration.

### VSSI-MS Measurements

The multidevice setup (Figure S1) was coupled to a linear ion trap mass spectrometer (LTQ-XL, Thermo Fisher, San Jose, CA, USA). Analyte and D_2_O solutions were infused through PTFE tubing connected to a fused-silica capillary glued to the respective (analyte or D_2_O) emitter tip. For HDX-MS experiments, a flow rate of 10 μL/min for both analyte and D_2_O solutions was used employing a syringe pump (KD Scientific, Sigma-Aldrich, St. Louis). The analyte-containing emitter tip was aligned end-on with respect to the MS inlet (5 mm apart).^34^ The D_2_O emitter was maintained perpendicular to the analyte device and spaced ∼1 mm from the MS inlet. External bias voltages of +1.6 kV and +300 V were applied via platinum wires inserted into the PTFE tubing for the analyte and D_2_O devices, respectively.

Replicate mass spectra were recorded for the different peptides. Between replicates, the RF voltage supplying the piezoelectric transducers was removed as well as the DC bias voltage. These voltages were then reapplied prior to data collection for each replicate. For the peptides PA, PS, Nt17, BK, and KDD, the number of separate mass spectra generated for HDX analysis are 7, 6, 9, 6, and 8, respectively. Between data collection of individual spectra, the RF and DC voltages were turned off and the position of the emitter tips adjusted very slightly to try and achieve greater levels of HDX. Micropositioning in this fashion produced the wide range of reagent exposures shown in Figures 1 and S3. Differences in droplet plume density directly at the capillary entrance of the mass spectrometer (especially the D_2_O reagent) were responsible for the observed exposure differences. Based on the production of different charge states for the peptides, the numbers of separate HDX measurements for computing deuterium uptake for the respective peptides are 14, 12, 18, 12, and 21 (see Figure 1).

### MD Simulations

All-atom MD simulations where each analyte in periodic water box conditions were employed. The detailed setup for MD simulations is provided in Table S4. Initially, a MD system was developed for KDD (Pep-Fold 3.5 source),^77^ BK (pdb id: 6f3v), PA, Nt17 (pdb id: 3IOW, seq: 371–387), and PS. The VMD Molefacture molecule builder plugin was used to generate a helical structure for PS and PA. For MD studies, visual molecular dynamics (VMD) was used for visualization as well as a data analysis tool using both text and display modes.^78^ NAnoscale molecular dynamics (NAMD)-2.15 was used as the MD engine for all simulations presented here.^79^ The CHARMM36 force field (FF) parameter set was used for all simulations of the peptides hence the atom names were converted to CHARMM FF compatible names using the online CHARMM GUI input generator (CHARMM-GUI).^80−82^ Next, the solvate1.7 VMD plugin was used to add a cubic periodic water box with edges of 7 nm and electrostatics were neutralized by 150 mM NaCl. The starting structures for each peptide were random coil conformation. To attain these starting unstructured forms, high temperature simulations in *NPT* ensemble at 373, 473, and 673 K were conducted. The CHARMM36 FF parametrization was used for the peptides and small ions, and the pre-equilibrated TIP3P water model was used for the solvent molecules.^83,84^ Each high-temperature simulation was completed in three steps, namely: (1) minimization for 10,000 steps; (2) equilibration at 0 K for 0.5 ns; (3) temperature ramping (10,000 steps of 1 fs going from 1 K to the higher temperatures–see above); and, (4) subsequent simulation for 2.5 ns with a 1 fs time step. In *NPT* ensemble, temperature and pressure were kept constant using Langevin dynamics and Nose–Hoover Langevin piston, respectively. A pdb structure (conformers unstructured) was generated from the high-temperature simulation. Here the omega dihedral angle distribution showed coherent structural integrity of each peptide (no cis/trans isomerization) as shown in Figure S15. Then, the solvate 1.7 plugin was again used to generate a water box with the appropriate *x*, *y*, and *z* dimensions, and the PSFGEN package with the CHARMM36 general FF (cgenff.rtf) topology was used to generate the initial psf and pdb files.

Throughout the MD simulations, the CHARMM36 FF parameter set was used for water, ions, and analytes.^85^ Before performing energy minimization and equilibration at 300 K, the structural integrity of each peptide was examined. Simulations were run in four steps as follows. After running 10,000 step minimizations, all systems were equilibrated in two steps. First, heavy atoms were maintained at constant volume to equilibrate water molecules at 0 K. Then the temperature was slowly raised from 0 to 300 K and a 20 ns equilibration was performed at *NPT* ensemble (*P* = 1.103 atm, *T* = 300 K). Finally, a production run was executed for 500 ns for each peptide comprising ≥3 replicates. All production runs were conducted in an *NPT* ensemble keeping Langevin thermostat and Nose–Hoover Langevin barostat constant using a 2 fs time step. The electrostatics and Van Der Waals interaction cut off was 12 Å with the particle mesh Ewald technique (PME) maintaining the Langevin thermostat at 300 K. Overall, the number of replicate MD trajectories for peptides PA, PS, Nt17, BK, and KDD were 3, 3, 3, 6, and 6, respectively. The larger number of replicates for the smaller peptides were required to obtain similar structure convergence.

### Data Analysis

All analyses were performed using TCl/Tk, BASH, Python, and R, scripts. The python package SOURSOPS was used to generate the *R*_g_ versus δ plots which provides secondary structure information in the form of the most probable conformations in solution.^86^ Data visualization employed Matplotlib and Seaborn; the secondary structure analysis was carried out using VMD and visualized with Seaborn.^78^ Analysis and visualization of MD simulations used a combination of tcl scripts, the VMD timeline plugin,^87^ and python scripts. The Xcalibar (Thermo, Pittsburgh, PA) software suite was used to extract mass spectral data which was exported to Excel (Microsoft, Redmond, CA) which was used for further analysis and figure production.

## Conclusions

In-droplet HDX measurements have been conducted for five peptides representing a wide range of structural flexibility and amide hydrogen sites. MD simulations experiments have been used to develop an HDX reactivity-to-structure calibration curve. The new model considers the accessibility of different sites obtained from MD simulations scaled by differences in experimentally determined *k*_int_. At this early stage the model is relatively robust showing an ability to provide a strong correlation between experimental and theoretical % BB exchange across the highly variable peptide structure landscape.

New insights about the structural flexibility and structural bias of disordered peptides have been elucidated from the MD simulations. First, the peptide BK exhibits somewhat limited flexibility. This may impart decreased access to backbone amide hydrogens not currently captured well in the model. Second, the MD simulations suggest some conformer inflexibility for Nt17 resulting from rapid helix formation/deformation across the mid region of this α-MoRF peptide. Finally, the study suggests that the model may be used to provide a scale for in-droplet HDX levels revealing information about IDR structural flexibility and bias.

Given the prevalence of IDPs within eukaryotic organisms, the development of foundational work toward a structure prediction model is timely. The simplicity—yet enhancements—afforded by the VSSI technology provides a roadmap for developing high-throughput structural screening capabilities with HDX-MS to enhance drug and biomarker discovery efforts in the future.

## Data Availability

All mass spectral data sets are available as.RAW files on Google Drive supported by WVU. Representative MD trajectory files are also located on this repository. Additionally, all excel spreadsheets used to plot the data are available as well as scripts for calculating the open and closed states of different peptide structures. The files can be downloaded from https://drive.google.com/drive/folders/18cuIdVUFpcmiiM25JJFwF4amaIgi1g4X?usp=sharing.

## Supporting Information Available

Fifteen figures showing instrument setup, MS data, correlation data, and MD simulations results. Four tables showing multiple regression results, correlation statistics, and MD simulations setup. HDX calculation section. Error calculations section (PDF)
