# Supplementary material for: Structure Characterization of a Disordered Peptide Using In-Droplet Hydrogen/Deuterium Exchange Mass Spectrometry and Molecular Dynamics
Source: ACS Phys Chem Au. 2024 Nov 13;5(1):17–29. doi: 10.1021/acsphyschemau.4c00048 (PMC11758492; doi:10.1021/acsphyschemau.4c00048)
Supplement: Supplementary file 1 — pg4c00048_si_001.pdf [file pg4c00048_si_001.pdf]

Supporting Information for:

Structure Characterization of a Disordered Peptide Using In-Droplet Hydrogen/Deuterium Exchange Mass Spectrometry and Molecular Dynamics

Mohammad A. Rahman,[1] Mst Nigar Sultana,[1] Daud Sharif,[1] Sultan Mahmud,[1] Justin Legleiter,[2] Peng Li,[1] Blake Mertz,[3]* Stephen J. Valentine[1]*

[1]Department of Chemistry, West Virginia University, Morgantown, WV 26506, USA
[2]Department of Biochemistry & Molecular Biology, University of Nevada, Reno, Reno, NV 89557, USA
[3]Alivexis, Cambridge, MA 02142, USA
*mertzjb@gmail.com and *stephen.valentine@mail.wvu.edu

TABLE OF CONTENTS

Figure S1. Photograph of the dual emitter VSSI setup used in these studies……….……………..2

Figure S2. Representative HDX-MS data for amino acids…………………………………………3

Figure S3. Linear regression plots for amino acids…………………………………………………4

Table S1. Linear regression statistics for amino acids………………………………………………5

Table S2. Hydrogen type reactivity propensity values………………………………………………6

Figure S4. Representative HDX-MS data for peptides……………………………………………...7

Table S3. Linear regression statistics for peptides…………………………………………………...8

HDX Calculation……………………………..…………………………………………………………9

Error Calculations……………………………………………………………………………………10

Figure S5. Plot of secondary structure propensity versus MD time for PA peptide……………...11

Figure S6. Plot of secondary structure propensity versus MD time for BK peptide……………..12

Figure S7. Plot of secondary structure propensity versus MD time for KDD peptide…………...13

Figure S8. Plot of secondary structure propensity versus MD time for PS peptide……………..14

Figure S9. Backbone RMSD plots for MD trajectories for different peptides……………..……...15

Figure S10. Asphericity versus radius of gyration plots for different peptides………...………16

Figure S11. Contact map distributions for different peptides………………………………………17

Figure S12. Plot of secondary structure propensity versus MD time for Nt17 peptide…………18

Figure S13. Contact map distributions for Nt17 peptide……………………………………………19

Figure S14. Asphericity versus radius of gyration plot for Nt17 peptide…………………………..20

Table S4. Summary of molecular dynamics simulations setup……………………………………21

Figure S15. Omega dihedral angle distribution for the different peptides………………………..22

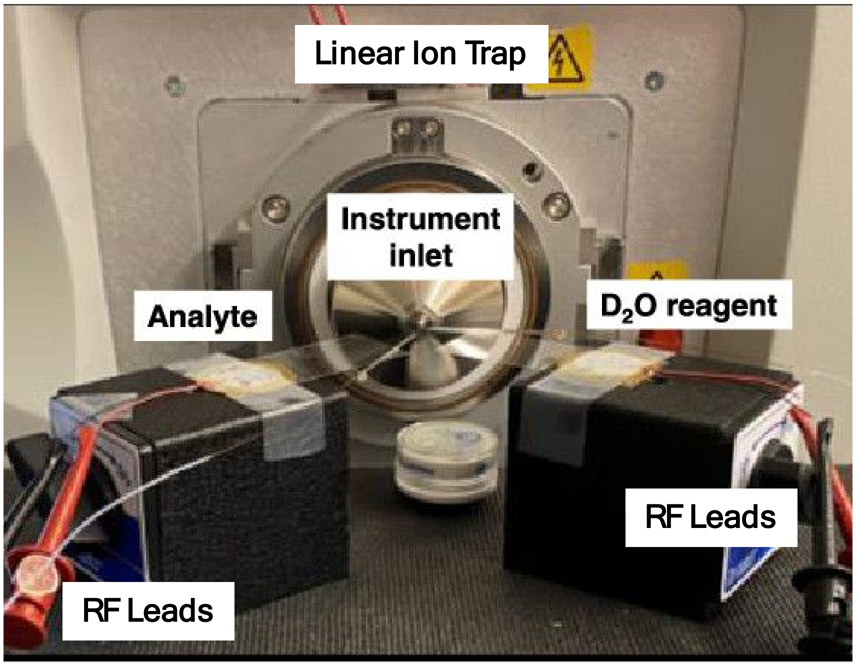

Figure S1. Photograph of the dual emitter VSSI experimental setup. Shown are VSSI devices (piezoelectric buzzer, microscope slide, and pulled emitter tip). The devices associated with analyte and reagent droplet plume production are labeled. The mass spectrometer and its inlet are also labeled. The RF power source for the transducers are also labeled.

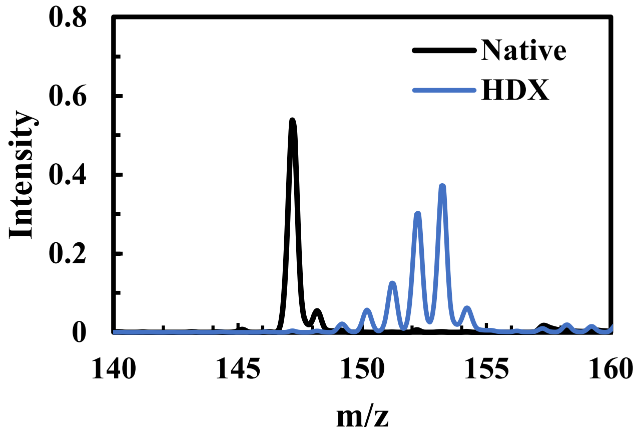

Figure S2. Representative HDX cVSSI-MS data for [M+H]+ K ions. A zoomed-in region of the mass spectrum shows the intensity vs m/z. The shift in isotopic distribution is revealed with traces of different color. Here the absence and presence of D_2O reagent is presented by black and blue traces, respectively.

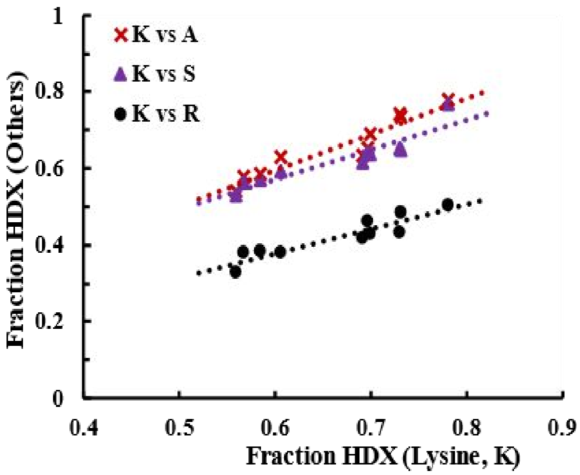

Figure S3. Linear regression plots for amino acid standards. Shown are results for the K vs R (circles), K vs A (cross), and K vs S (triangle) relationships. Dotted lines: best-fit lines for each respective relationship. These data were collected for different exposure conditions (ratio of D_2O to H_2O) as evidenced by differences in fraction HDX for the internal standard, K.

Table S1. Linear regression analysis for HDX behavior of individual amino acid residues

**K ($[M+H]^+$) Vs**	***m***[a]	**b**[b]	**R^2**	**Error y**[e]	**SS_reg**[f]	**SS_res**[g]
A	1.1(0.1)	-0.03(0.09)	0.91	0.03	60.47	6
S	0.9(0.1)	0.05(0.10)	0.86	0.03	36.44	6
R	0.64(0.09)	-0.01(0.06)	0.85	0.02	47.09	6

[a]Slope of the best-fit line through the data obtained using the LINEST function in Excel. The error in the slope as reported by LINEST is provided parenthetically.
[b]*y*-intercept of the best-fit line through the data obtained using the LINEST function. The error in the y-intercept as reported by LINEST is provided parenthetically.
[e]Standard error of the mean for the y estimate from the LINEST function.
[f]Regression sum of squares from the LINEST function.
[g]Residual sum of squares from the LINEST function.

Table S2. Hydrogen type HDX propensity values obtained from the gradient descent machine learning optimization.

N_t[a]	R[b]	K[c]	S[d]	C_t[e]
0.7877[f]	0.3082	0.7722	0.5630	0.6782

[a]N-terminal hydrogen
[b]Arginine sidechain hydrogen
[c]Lysine sidechain hydrogen
[d]Serine sidechain hydrogen
[e]C-terminal hydrogen
[f]HDX propensity factors were calculated for the exchangeable hydrogens of side-chain residues using nonnegative linear regression in MATLAB. First a $n \times m$ matrix X was entered into MATLAB. Here n and m are the total number of hydrogen types (in the table above) for each amino acid residue and the number of total HDX measurements, respectively. Each amino acid residue was measured 30 times so X was a 5×30 matrix. Second, a 1×30 vector (Y) containing the deuterium uptake of the separate amino acid residues was entered into MATLAB. The total exchange values were scaled to 75% exchange levels using the slopes given in **Table S1**. Next the command b = lsqnonneg(X,Y) was performed from the command prompt and the HDX propensity values were generated. The validity of this approach was cross checked using a separate approach to verify that similar HDX propensity values could be obtained. Here, with a home-built python script a system of linear equations (SLE) was constructed from separate HDX experiments employing four amino acid residues (i.e., K, A, S, R). A gradient descent machine learning algorithm was used to estimate propensity parameters by setting optimization criteria using **Equation S1**:

$$Ax = b \qquad S.$$

In **Equation S1**, A is a matrix of coefficients, x is the vector of unknown parameters, and b is the experimental deuterium incorporation. The system of equations was solved for the unknown x parameters using gradient descent by optimizing the cost function $J(x)$ that quantifies the error between the left- and right-hand sides of Equation S1. $J(x)$ is defined by **Equation S2**:

$$J(x) = ||Ax - b||^2 \qquad S.$$

Here, ||.|| represents the Euclidean descent and the goal is to minimize $J(x)$ with respect to x iteratively until convergence. The process is described by **Equation S3**:

$$x(k+1) = x(k) - \alpha \nabla J(x(k)) \qquad S.$$

In **Equation S3**, $x(k)$ are estimates of the solution, $x(k+1)$ is the updated solution, α is the learning rate, and $\nabla J(x(k)$ is the gradient of the cost function. The gradient was calculated as $\nabla J(x(k) = 2A^{T(Ax-b)}$. Iterations were performed until the minimization was completed. For this analysis the matrix was constructed using the following criteria. The maximum achievable HDX was determined to be 86% based on that obtained for the internal standard K. Therefore, for each HDX experiment, the % deuterium incorporated for K was scaled to achieve 86%. The scaling factor was then applied to the % deuterium uptake for the S, A, and R amino acids standards. HDX propensity parameters were computed for 5 different types of heteroatom sites: charged and neutral N terminal hydrogens (3 or 2), C-terminal hydrogen (1), K side-chain hydrogens (3), serine hydroxyl hydrogen (1), and arginine guanidino hydrogens (5). These are common to both the amino acid standards and the peptides in this study.

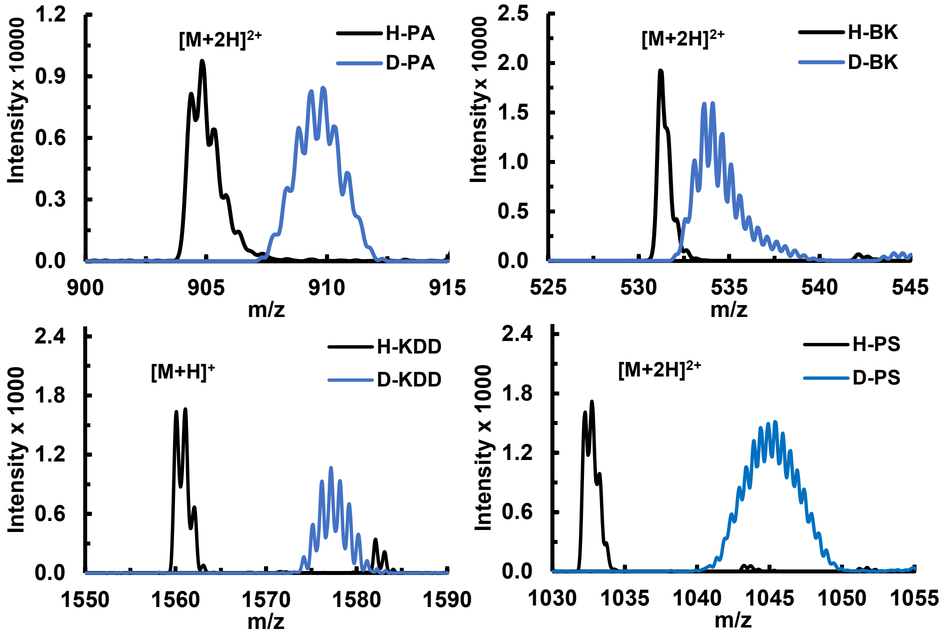

Figure S4. Representative mass spectral data for the different peptide systems. Shown are zoomed in regions presenting the relative shifts in m/z of each analyte (M) including BK (top right), PA (top left), KDD (bottom left), and PS (bottom right) before and after undergoing HDX. Black and Blue traces represent the absence of D_2O (H-M) and the presence of D_2O (D-M) reagent, respectively. Peptide ions are produced from native solutions and exposed to in-droplet HDX reactions (see Experimental section for details). Replicate experiments (n≥6) were performed to obtain average exchange values (**Table 1**). *m*/*z* shifts were used to compute the deuterium uptake for each peptide which was plotted against that for the internal standard K as shown in **Figure 1**. The statistical values for best-fit lines for these plots are provided in **Table S3**.

Table S3. Linear regression analysis for the peptide data shown in Figure 1

Entry		m^a	b^a	R^2	Error y^c	SS_reg[d]	SS_res[e]
PA	$[M+3H]^{3+}$	0.34(0.08)	2.94(5.03)	0.80	1.09	24.24	5.94
	$[M+2H]^{2+}$	0.35(0.06)	2.65(4.29)	0.86	0.93	26.40	4.31
KDD	$[M+3H]^{3+}$	0.73(0.03)	-4.51(1.70)	0.99	1.38	1615.99	11.47
	$[M+2H]^{2+}$	0.67(0.03)	-1.63(1.82)	0.99	1.48	1353.51	13.15
	$[M+H]^{+}$	0.75(0.05)	-7.44(3.56)	0.98	1.27	424.45	8.03
Nt17	$[M+2H]^{2+}$	0.39(0.07)	8.02(4.74)	0.82	1.24	49.82	10.69
	$[M+H]^{+}$	0.43(0.08)	7.02(5.83)	0.78	1.52	58.79	16.16
BK	$[M+2H]^{2+}$	0.20(0.05)	18.90(3.57)	0.80	1.12	20.33	5.03
	$[M+H]^{]+}$	0.43(0.08)	1.64(5.41)	0.89	1.70	90.01	11.56
PS	$[M+3H]^{3+}$	0.68(0.11)	-2.68(7.69)	0.91	1.00	40.48	4.03
	$[M+2H]^{2+}$	0.68(0.09)	1.85(6.71)	0.93	0.88	40.48	3.06

[a]Slope of the best-fit line through the data obtained using the LINEST function in Excel. The error in the slope is provided parenthetically and is also obtained from the LINEST function.
[b]*y*-intercept of the best-fit line through the data obtained using the LINEST function. The error in the y-intercept is provided parenthetically and is also obtained from the LINEST function.
[c]Standard error of the mean (see Methods section) for the y estimate from the LINEST function
[d]Regression sum of squares from the LINEST function
[e]Residual sum of squares from the LINEST function

HDX Calculation

Here, the calculations required to obtain %BB exchange are provided for $[M+2H]^{2+}$ PA ions shown in Figure S4.

First, the total amount of deuterium incorporated is determined. As shown in Figure S4, the unreacted doubly-charged ions have an average *m*/*z* of 905.0. The reacted ions have an average *m*/*z* of 909.4. Because these are doubly-charged ions, the amount of incorporated deuterium is $\Delta m/z \times z$ or 4.4 × 2 (8.8). This corresponds to ~28% deuterium uptake under these conditions. Multiple exchange values like this were plotted against the percent deuterium incorporation for the internal standard K (see Figure 1). Using the regression statistics for doubly-charged PA ions (Table S3), it is possible to obtain an estimated exchange level for doubly-charged PA ions at 75% exchange for K. Here, the regression provides a peptide exchange value of y = mx + b = 0.35(75)+2.65 (Table S3). The on average deuterium uptake scaled to 75% internal standard exchange is thus 28.9% for doubly-charged PA ions. That is, on average, these ions would be expected to exchange 0.289×31 (29 exchangeable hydrogens + 2 protons). So, on average doubly charged PA ions would be expected to exchange 8.959 hydrogens.

To then calculate the %BB exchange, it is necessary to first estimate the amount of the total exchange that would be expected to occur on the side chains under similar exchange conditions. Here, the parameters shown in Table S1 are used. The PS peptide has 4 K residues. Additionally, it is assumed that the extra protons are on K residues. Thus, the total number of exchangeable side chain sites arising from K is 4×2+2 = 10. The estimated deuterium uptake by these sites is this number multiplied by the propensity factor for K 10×0.7722 = 7.722. In addition to these fast-exchanging hydrogens, there is 1 carboxy terminus hydrogen (1×0.6782 = 0.6782). Thus, the total estimated side chain uptake is 7.722 + 0.6782 = 8.4 for this doubly-charged ion. Thus, the average deuterium uptake by the PA peptide backbone is thus 8.9590 – 8.4002 = 0.5588. The %BB exchange then is computed to be 0.5588 / 20 * 100% = ~2.8%. Because multiple doubly- and triply-charged ions were used to create the correlation plots shown in Figure 1, the average %BB exchange scaled at 76% internal standard K exchange for all ions can be obtained using a weighted average of estimated doubly- and triply-charged ion exchange. This is the estimated exchange value provided in Table 1 in the text. The uncertainties can be obtained by propagation of uncertainties in the estimated average values for doubly- and triply-charged values. These separate uncertainties are obtained by propagation of errors in m and b values from the best fit lines (Table S3) as outlined in Error Calculation below. Average values for deuterium uptake, total numbers of sidechain and backbone hydrogens in the neutral peptides, estimated side chain contributions, and %BB exchange for each peptide are provided in Table 1 in the text.

Error Calculations

All reported errors associated with slope and intercept values for best-fit lines obtained from the LINEST function in Excel can be represented by the equations presented below. For the slope error, the error is defined as the standard error of the slope (se_m) and can be expressed as:

$$se_m = \sqrt{\frac{s_e^2}{\sum (x_i - \bar{x})^2}} \qquad S4.$$

In **Equation S4**, x_i and $\bar{x}$ represent the *i*th *x* value and the mean of the *x* values. The term s_e^2 is the mean squared error of regression and is defined in **Equation S5** as:

$$s_e^2 = \frac{\sum (y_i - \hat{y}_i)^2}{n-2} \qquad S5,$$

where y_i, $\hat{y}_i$, and *n* represent the *i*th *y* value, the *i*th predicted *y* value, and the number of points in the determination, respectively. The standard error of the intercept (se_b) can be expressed as (**Equation S6**):

$$se_b = \sqrt{s_e^2\left(\frac{1}{n} + \frac{\bar{x}^2}{\sum (x_i - \bar{x})^2}\right)} \qquad S6.$$

Uncertainties in peptide average deuterium uptake shown in Table 1 are obtained through error propagation. First the uncertainty in the estimated deuterium uptake for each charge state is propagated from the error in the m and b variables obtained from the LINEST function in Excel. For the formula y = mx + b, this error propagation has the functional form shown in **Equation S7**.

$$[\varepsilon(y)]^2 = \left(\frac{\partial y}{\partial m}\right)^2 [\varepsilon(m)]^2 + \left(\frac{\partial y}{\partial b}\right)^2 [\varepsilon(b)]^2 \qquad S7$$

In **Equation S7** $[\varepsilon(y)]^2$, $[\varepsilon(m)]^2$, and $[\varepsilon(b)]^2$ correspond with the squares of the error in the estimated % total exchange, slope, and intercept, respectively. The latter two errors are found in Table S3. Next the separate uncertainties for the different charge states are further propagated using the same equational form (**Equation S7**) but for the weighted average formula for estimating the exchange for all charge states. Finally, the uncertainties in %BB exchange are populated in the same manner using the formula for estimating %BB exchange (see HDX calculation above). These uncertainties are also presented as the y-error bars in Figure 2.

For errors reported for %Error (Table 2) and x-error bars in Figure 2, the value represents the standard error of the mean ($\sigma_{\bar{x}}$) and is obtained from **Equation S8**:

$$\sigma_{\bar{x}} = \frac{\sigma}{\sqrt{n}} \qquad S8,$$

where $\bar{x}$ and σ represents the average measurement and the standard deviation, respectively.

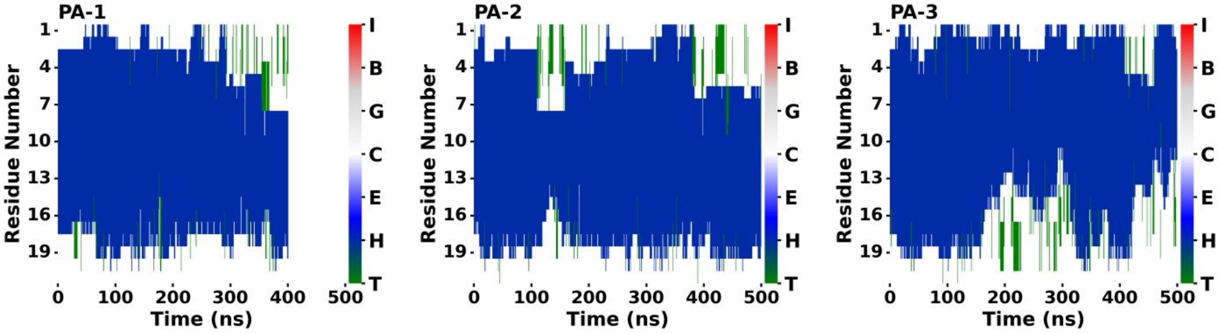

Figure S5. PA peptide secondary structure evolution across three 500-ns MD trajectories. Overall, PA primarily developed into two forms of secondary structure namely α-helix and extended helix at the two termini.

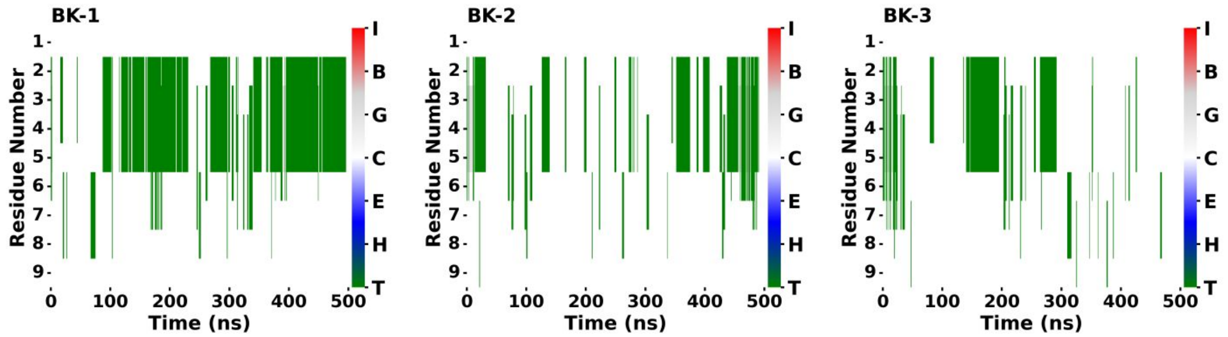

Figure S6. BK peptide secondary structure evolution across three separate 500 ns MD trajectories. Overall, BK primarily exhibited turn and coil conformations which ultimately resulted in structural inflexibility (see Results section for details).

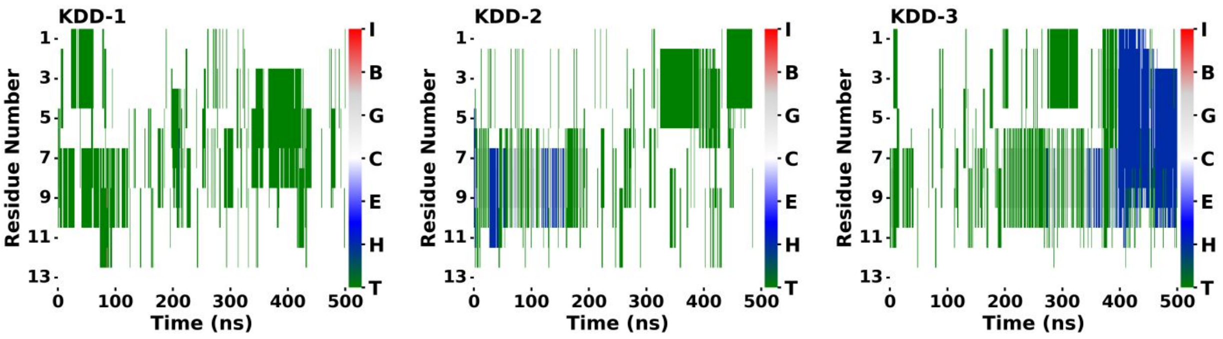

Figure S7. KDD peptide secondary structure evolution across four separate 500-ns MD trajectories. Overall, KDD primarily exhibited turn and coil conformations; however, residues 6-12 adopted very transient helical conformation periodically.

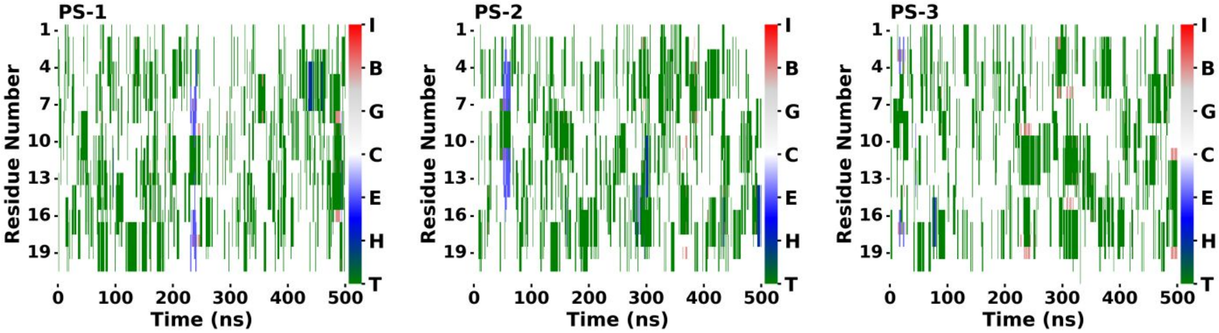

Figure S8. PS peptide secondary structure evolution across three separate 500-ns MD trajectories. Overall, PS primarily exhibited high flexibility with extensive coil conformation.

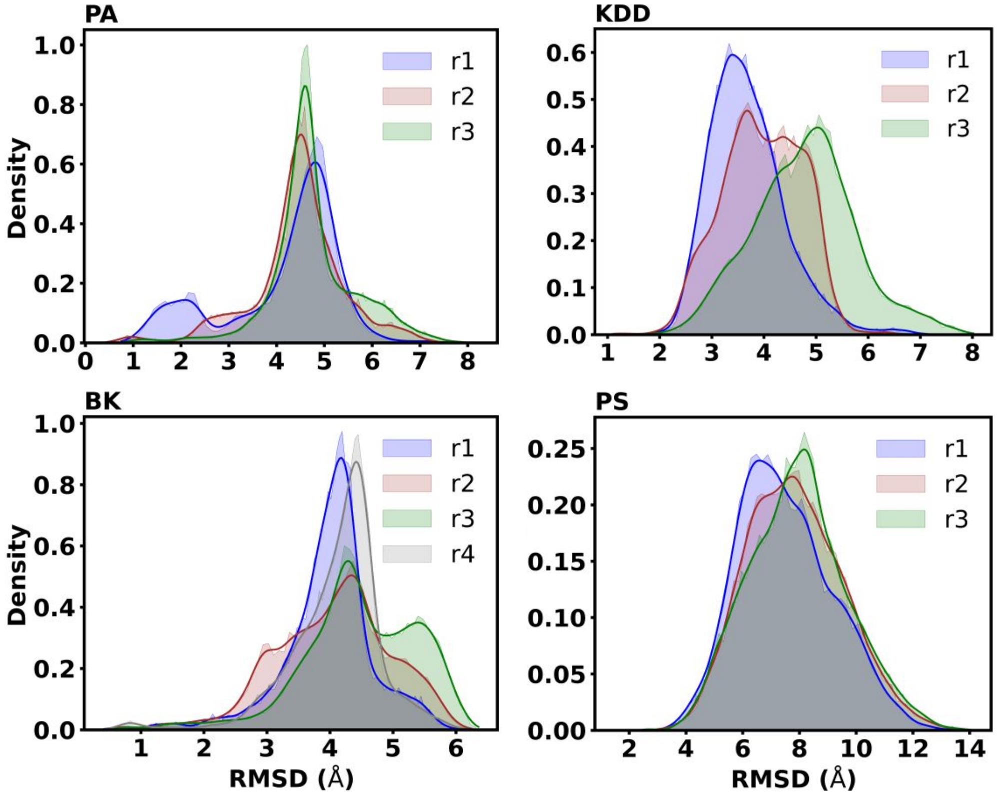

Figure S9. Backbone Root Mean Square Deviation (RMSD) plots for the different peptide systems. Shown are data for PA (top left), BK (bottom left), KDD (top right), and PS (bottom right). Density (unitless) in the bottom left plot refers to the fraction of conformers having the RMSD value and r1, r2, r3, and r4 refer to separate MD trajectories. Notably, a similar trend to that observed for Figures S6 to S9 is obtained. That is, the greater the backbone RMSD, the more flexible the structure is. In-house TCL and python scripts have been used to generate the data and the plots, respectively.

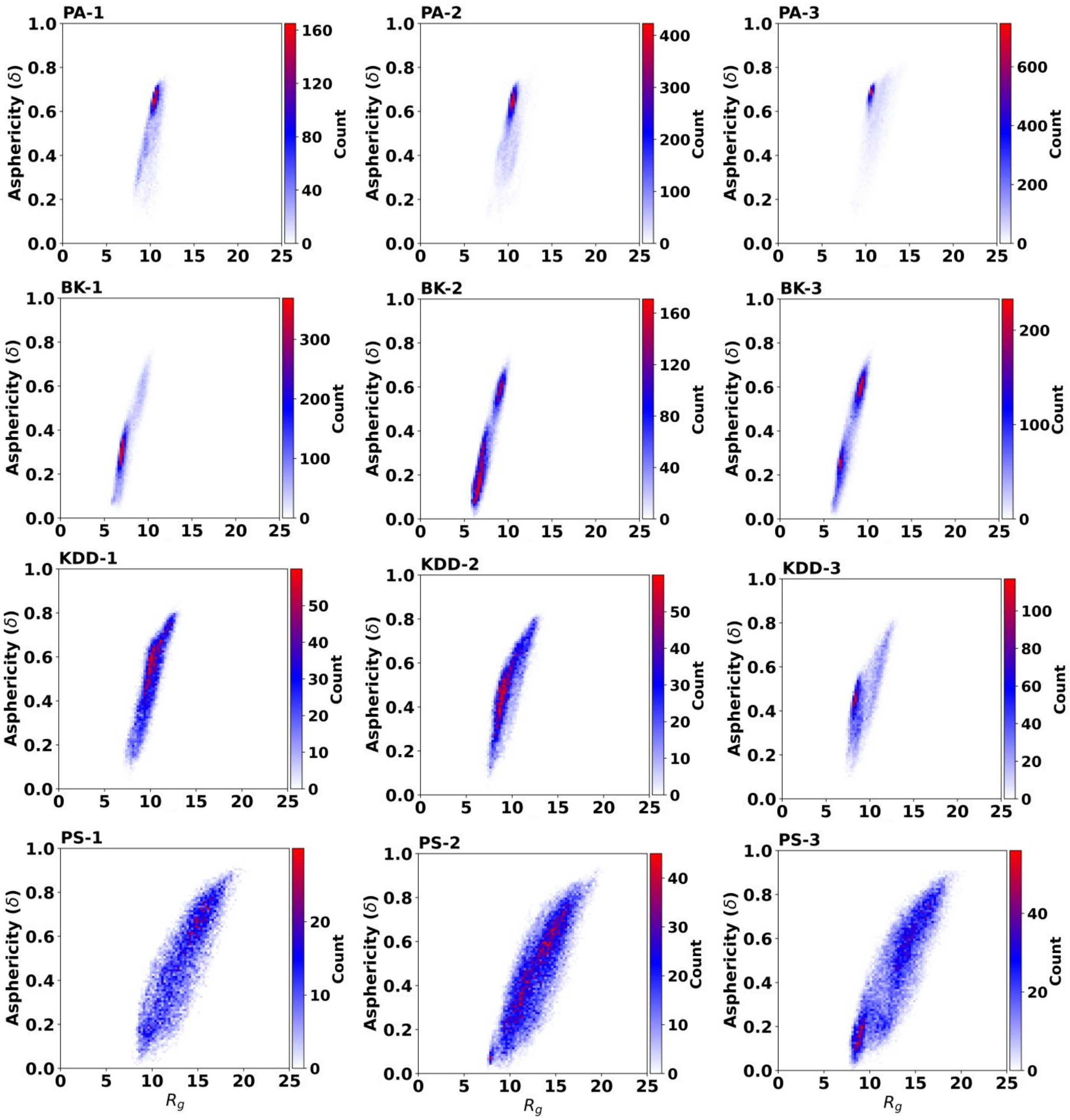

Figure S10. Asphericity (δ) Vs Radius of Gyration (R_g) for model peptides. δ versus R_g distributions describe the degree of structure fluctuation or structural flexibility for separate MD trajectories. Shown are data for PA (top row), BK (second row), KDD (third row), and PS (bottom row). Overall, PA exhibits the least conformational fluctuation with a relatively narrow distribution and a high-density region at R_g ~10 and δ ~ 6 to 8; this indicates secondary structure retention. The python package SOURSOPS was used to generate the δ versus R_g plots which provides secondary structure information in the form of the most probable conformations in solution[8].

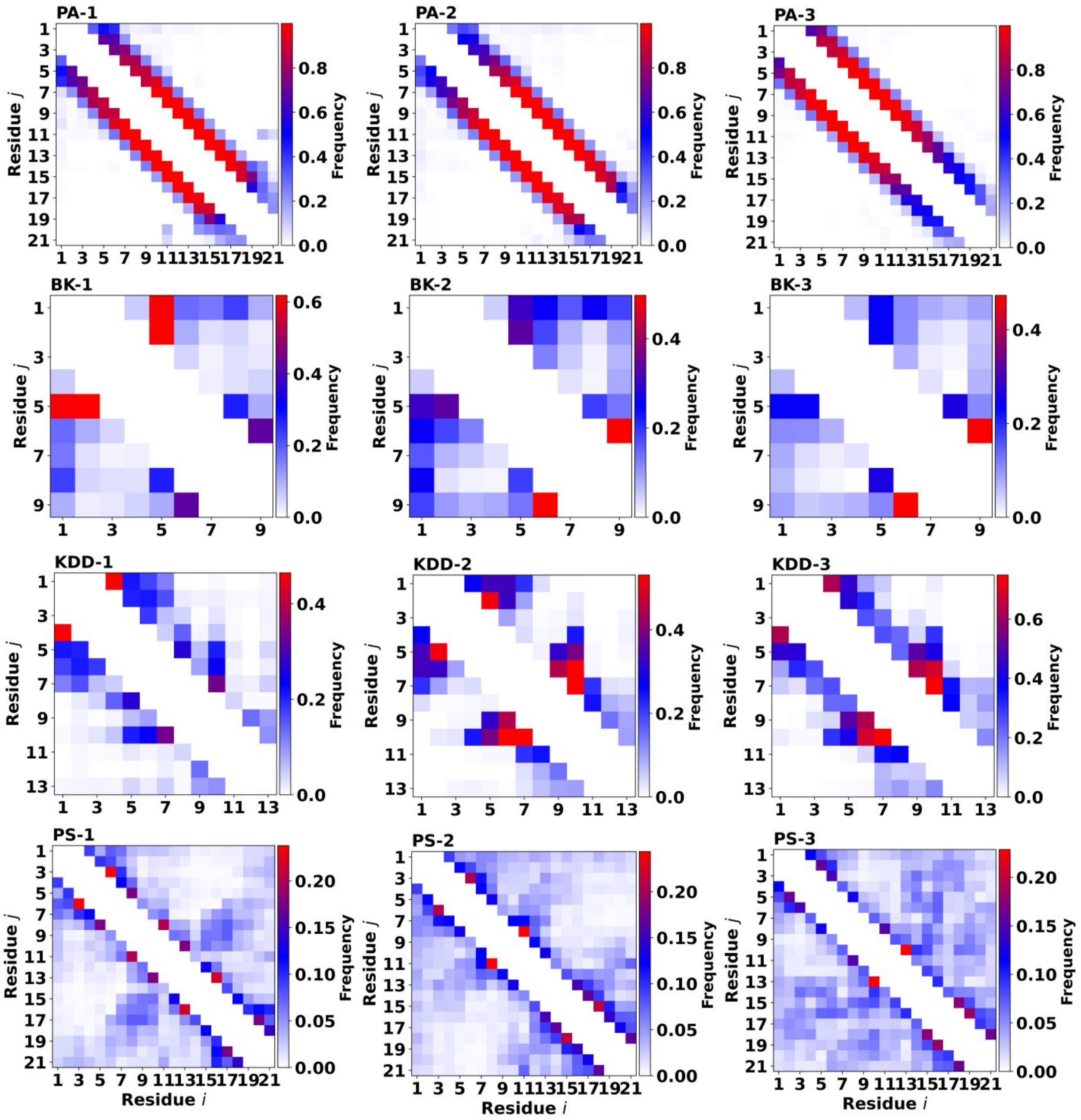

Figure S11. Contact map distributions [residue (*i*) vs residue (*j*)]. The plots (3 replicates) show the secondary structure formation tendency of PA (top row), BK (second row), KDD (third row), and PS (bottom row). The color bar (frequency) represents the degree of an event (*i* and *j* come within contact distance to form H bond). The MDTraj suite was used to generate the residue contact distribution in Jupyter Notebook.

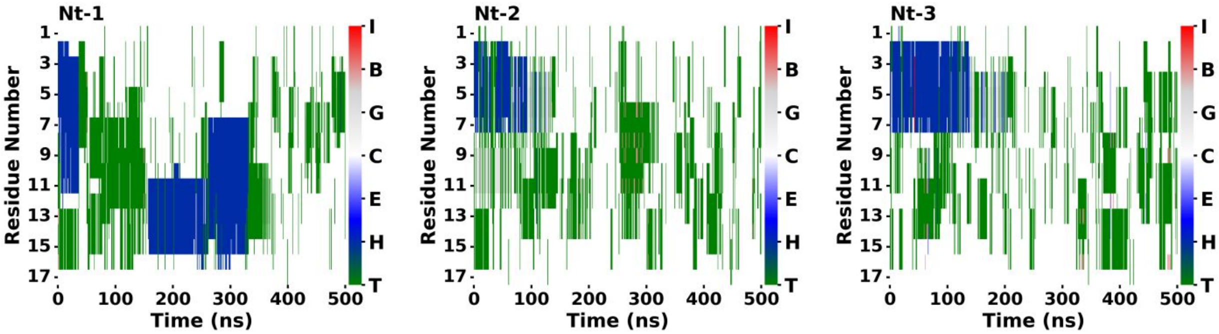

Figure S12. Nt17 peptide secondary structure evolution across three separate 500-ns MD trajectories. Overall, Nt17 primarily exhibited high flexibility with very transient helical conformation formation across the entire peptide and across the entire MD trajectories.

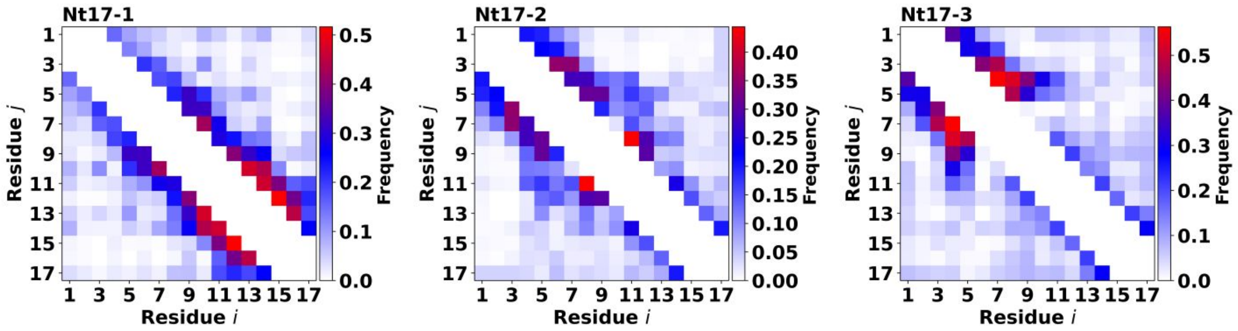

Figure S13. Contact map distributions [residue (*i*) vs residue (*j*)] for Nt17. The plots (3 replicates) show the secondary structure formation tendency of Nt17. The color bar represents the degree of an event (*i* and *j* come within contact distance to form H bond). The MDTraj suite was used to generate the residue contact distribution in Jupyter Notebook.

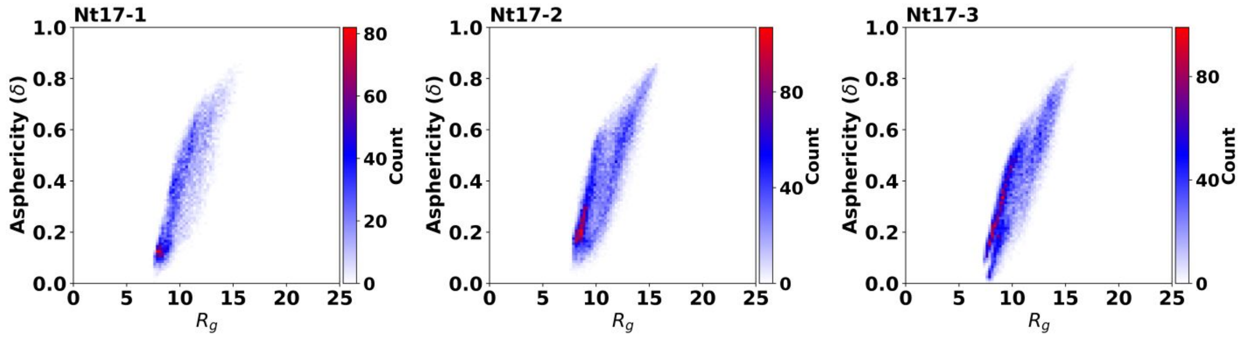

Figure S14. Radius of gyration (R_g) vs asphericity (δ) plots for Nt17 obtained from three 500-ns MD trajectories. Overall, the distributions are fairly broad suggesting a high degree of structural transformation. However, some density is observed at about R_g of 7 to 8 and δ ~ 0.1 to 0.2 suggesting the high frequency formation of helical regions imparting some structural inflexibility.

Table S4. Summary of simulations setup and production run to build HDX model of different secondary structural elements and sequences.

Entry	Structure obtained	Initial Structure[a]	NVT (573K)	Minimization	Equilibration1, NPT (1 atm, 0 K) /ns	Equilibration2, NPT (1 atm, 300 K) /ns	Production /ns (3 run)
PA	VMD	H	RC	10000	2.5	20	500
Nt17	X-ray: 3IOW	H	RC	10000	2.5	20	500
BK	6f3b	RC	NA	10000	2.5	20	500
PS	VMD	H	RC	10000	2.5	20	500
KDD	Pep-Fold 3.5	RC-H	NA	10000	2.5	20	500

[a]H-Helix, RC-Random Coil, RC/H-mix of RC and H, NA-not applicable.

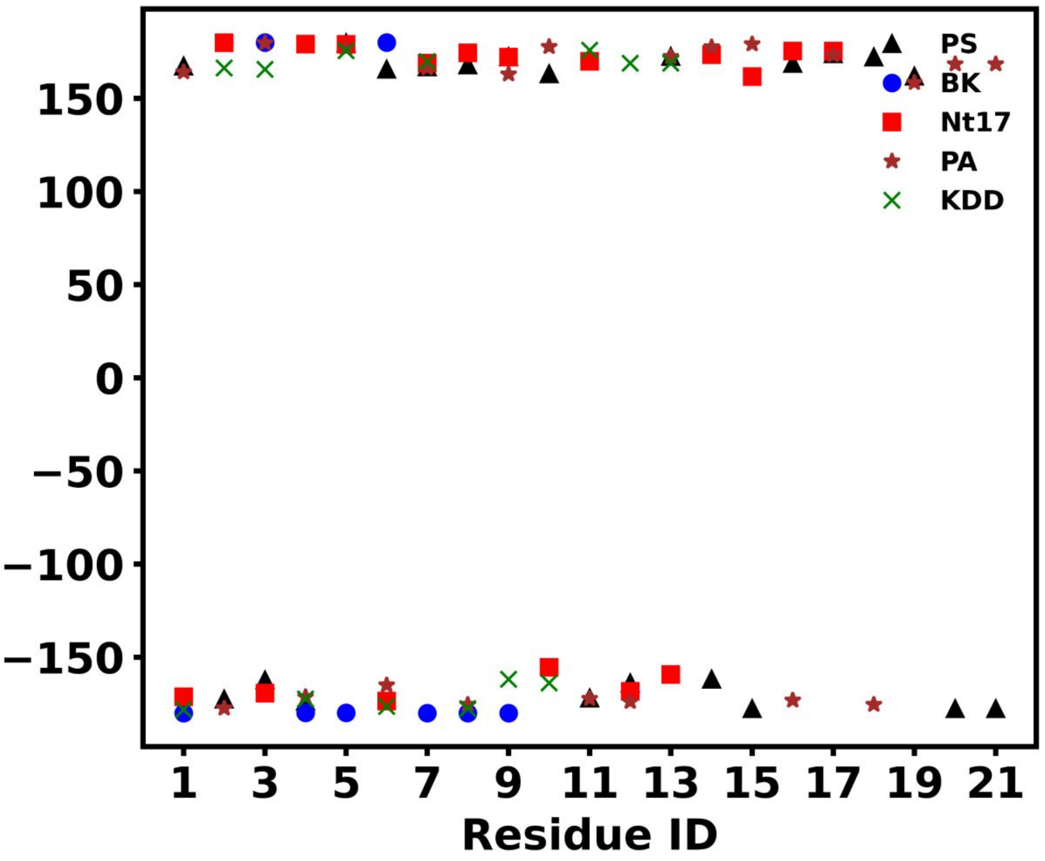

Figure S15. Omega dihedral angle distribution for the various peptides. The data shows that no structure transformation from cis to trans or trans to cis occurs. Data were collected from random coil structures of PS (black triangle), BK (blue circle), Nt17 (red square), PA (brown star), and KDD (green cross) peptides.
